# Eutrophication and sediment–water exchange of total petroleum hydrocarbons and heavy metals of Hashilan wetland, a national heritage in NW Iran

**DOI:** 10.1007/s11356-021-17937-x

**Published:** 2021-12-19

**Authors:** Sajjad Abbasi, Sara Sheikh Fakhradini, Neamatollah Jaafarzadeh, Pooria Ebrahimi, Shirin Yavar Ashayeri

**Affiliations:** 1 Department of Earth Sciences, College of Science, Shiraz University, 71454 Shiraz, Iran; 2 Department of Radiochemistry and Environmental Chemistry, Faculty of Chemistry, Maria Curie-Skłodowska University, 20-031 Lublin, Poland; 3 Environmental Technologies Research Center, Ahvaz Jundishapur University of Medical Sciences, Ahvaz, Iran; 4 Department of Earth, Environmental and Resources Sciences, University of Naples Federico II, 80126 Naples, Italy

**Keywords:** Heavy metal, TPH, Eutrophication, Wetland, Hashilan, Iran

## Abstract

The heavy metal(loid)s concentrations in water and sediments were analyzed in the Hashilan wetland to assess the spatial distribution, pollution status, fate, partitioning, and ecological risk and also to identify the heavy metal(loid)s sources in sediments using PMF (Positive Matrix Factorization) and APCs-MLR (absolute principal component score-multiple linear regression) receptor models. According to the pollution indices, (Ni, Cu, Cr, Mo), and (Zn, Cr, and Cu) are considered the most important pollutants in sediments and water, respectively. Ni, Cr, and Cu are the main contributors to ecological risks in sediments of some stations. The potential ecological risk assessment proposed low ecological risk in water of the study area. Higher distribution coefficient (*K*_*p*_) values of Ni, Cr, Mn, Cu, Co, Pb, As, and Zn indicated the majority of these heavy metals present in the sediments; whereas, the majority of Cd concentration occurs in water. PMF and APCs-MLR results indicated the natural sources were the main factors affecting the concentrations of Ni, Cr, Zn, Al, Co, Fe, Pb, As, Cd and somewhat Cu. Mixed natural and agricultural activities are the main sources of Mo, and somewhat Cu. According to the results, there is low pollution of TPH (total petroleum hydrocarbons) in the sediment samples. Also, phosphate (PO_4_^2−^) and nitrate (NO_3_^−^) concentrations were below the recommended permissible limits at all sampling sites except the S8 station for NO_3_^−^.

**Supplementary Information:**

The online version contains supplementary material available at 10.1007/s11356-021-17937-x.

## Introduction

Wetlands are recognized to be productive ecosystems due to water quality protection, sinks for nutrients and pollutants, water cycling maintenance, controlling floods and flows, groundwater recharge, and so on (Andreu et al. 2016; Tuboi et al. 2018). Nowadays, anthropogenic stressors such as population growth, resources overexploitation, economic development, and agricultural encroachments and drainage have affected and polluted the wetland environment (Tuboi et al. 2018). Metal Pollutants enter the aquatic habitats not only from natural sources including weathering, erosion, and soil leaching but also from anthropogenic activities such as urbanization, industrial and agricultural activities (Abbasi et al. 2019; Strady et al. 2017) and endanger humans and animals health as a result of their toxicity, persistence, and bioaccumulation via the food chain.

Total petroleum hydrocarbon (TPH) is a homogeneous mixture including hundred chemical compounds that originally come from crude oil. These compounds generally enter the aquatic environment through several anthropogenic activities such as contaminated lands, pesticides, vehicle emissions, oil spillage, automobile oils, or different harmful organic substances (Chen et al. 2020; Li et al. 2019). Several products of TPH contain heavy metals which are present in crude oil or may also be added as chemicals to these products (Defarge et al. 2018). However, numerous pesticides contain As, Co, Cr, Ni, and Pb elements and Cd, Co, Cu, Ni, Pb, and Zn metals as impurities are found in different fertilizers. Moreover, Fe, Mn, Zn, Pb, and Ni are present in some herbicides (Gimeno-García et al. 1996). Eating, breathing, and direct contact with contaminated soil, water, and sediments are considered the path of their entry into the human body (Quiñonez-Plaza et al. 2017). Also, these compounds can create negative effects on human and biota health, depending on the different compounds values present in the TPH (Quiñonez-Plaza et al. 2017; Zhou et al. 2014).

Sediments as a part of aquatic environments have a significant role in biochemical cycling and food web protection (Islam et al. 2018). Sediments provide a large capacity to accumulate a large fraction of heavy metal(loid)s as a result of hydrolysis, co-precipitation, and adsorption processes while a small portion of heavy metals remains in the water column (Hou et al. 2013; Wu et al. 2017). Also, sediments could act as a secondary source due to environmental conditions change and releasing metals into the water, affecting a health risk to aquatic biota due to their high bioavailability (Cui et al. 2019). However, metal(loid) partitioning between water and sediment is specified due to oxidation/reduction and precipitation/dissolution reactions of heavy metal(loid)s in sediments (Duarte et al. 2014).

Water resources are essential in sustainable societal development and natural processes including erosion and climatic conditions, and anthropogenic activities, such as industrial and agricultural practices affect water resources and a large amount of pollutants is discharged into the water reservoirs (Lyu et al. 2021). Eutrophication is considered as an important environmental problem in the world. This phenomenon occurs under the influence of natural factors and human activities, which increase the amount of algae and aquatic plants and decrease the dissolved oxygen of the water (Lin et al. 2020, 2021). Thus, the evaluation of water quality is a significant prerequisite for the conservation, control of water bodies, and safety guaranty of the regional water environment.

This study focuses on Hashilan wetland, which is one of the most important agricultural areas in Kermanshah province as a result of fertile soils, sufficient water resources and suitable climate. Also, Hashilan wetland is one of the unique flora and fauna regions of this Province. Unfortunately, discharge of agricultural runoff, containing different types of contaminants such as microplastic particles (Abbasi 2021), heavy metals, fertilizers, and herbicides into the wetland has affected human and biota health. Also, the presence of tourists in this area can also be a threat to this wetland. Thus, decision-makers need to evaluate heavy metal(loid)s values in water and sediment environments to specify the sources, fate, and ecological risk of pollutants and then eliminate water and sediment pollution via urgent actions.

Worth mentioning that the novelty of the current study is to investigate organic and inorganic contaminants comprehensively in the water and sediment of Hashilan Wetland for the first time. The major purposes of this research are 1) to specify the concentration, distribution, pollution rate and ecological risk of heavy metal(loid)s in water, and sediment the Hashilan wetland, 2) to determine the metal(loid)s source apportionment in the sediment, 3) to identify affective factors on metal(loid)s partitioning between surface water and sediment, 4) to evaluate the trophic state of the water samples, and 5) to investigate the pollution rate of TPH.

## Material and methods

### Study area

**Fig. 1:**
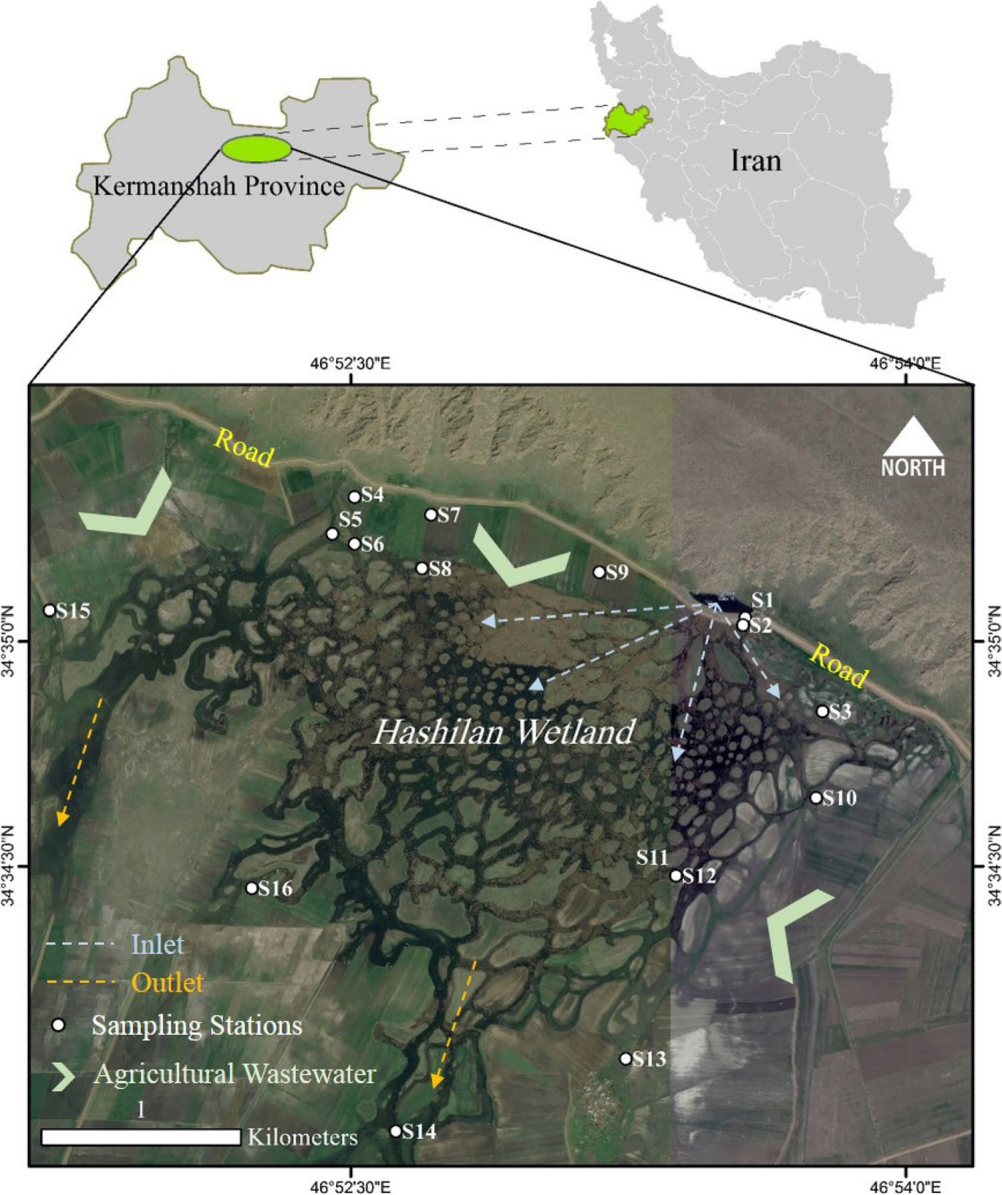
Map of the study area and sampling sites in Hashilan’s wetland area

Hashilan wetland, as a significant freshwater wetland in the Kermanshah Province, has been located at the Hashilan village, being 26 km northwest of Kermanshah city. It lies between 46°, 51′ to 46°, 54′ eastern longitude and 34°, 34′ to 35°, 34′ northern latitude (Fig. 1). This Wetland constitutes a unique feature in the Middle East which is called “karstic spring wetland,” an ecosystem mostly found in the Zagros Mountains of Iran. Geologically, its catchment basin and the aquifers of its water sources are located in the limestone formations of the Triassic-Jurassic age. Hashilan Wetland is located in a syncline bordered by two anticlines in the northeast (Khorin anticline) and southwest (Biston anticline). Surveying the wetland on a large scale is a part of the Miandarband plain which surrounds the two anticlines, contains several other wetlands and has two rivers namely Razavar in the north and Gareso in the south (Abbasi et al. 2021).

The mean annual precipitation is about 450 mm, respectively. Also, the average temperature is 22 °C. The areal coverage of wetland is about 450 ha. Sabz Ali spring, originating from the Khorein Mountains located on the northern border of the wetland supply the wetland water. This wetland consists of 110 islands ranging from 0.1 to 1 ha in size, which causes the water to be distributed among the islands as the small and big channels. This wetland has been suffered from high-intensity agricultural activities, which is the main occupation of the local inhabitants. It should be noted the wetland supports a significant and unique population of flora and fauna.

### Water and sediment sampling and analysis

Sixteen surface sediment samples and 6 water samples were collected in September 2019 covering an area of about 450 ha. In each sampling location, about 2 kg surface sediment (0–5 cm) was collected via a Van Veen stainless steel grab and placed in a clear polyethylene bag. Then, the samples were transferred to the laboratory in an iced box. It should be mentioned that the water samples were only taken from monitoring stations where the sediments were surrounded by water. The sampling stations information covering the whole study area is illustrated in Table S1 (supporting information).

In the laboratory, the sediment samples were air-dried at room temperature. Agate Mortar and pestle were used to crush the sediment. Larger debris such as plant leaves and pieces of glass were separated (2-mm sieve) after being air-dried at room temperature. Finally, the samples were passed through a 220 mesh (63 μm) for elemental analysis and then homogenized. Thus, the samples were measured using inductively coupled plasma mass spectrometry (ICP-MS) at Zarazma Mineral Studies Company, Iran.

Usually, sites close to roads indicate higher TPH contents than other areas. Therefore, to evaluate the TPH concentrations of Hashilan sediments, a total of 5 surface sediment samples were collected from stations close to the roads and agricultural lands where agricultural machinery passes and immediately put in dark glass bottles which were already washed with n-hexane and sealed by aluminum foil caps. The collected samples were transported to the laboratory of the Isfahan University of Technology in an ice-box at 4 °C and stored at -20 °C until analysis.

The surrogate standards (Pyrene-D10 lot:10,510 semi-volatile internal standards) and ultra-sonic bath (KUDOS Modell SK3210LHC) were used during the extraction of approximately 5 g per dried sample. For the TPH calibration, the pyr-D10 was used as the internal standard. The samples were then collected by means of a 30-ml organic solvent mix (DCM) of 1:1 v/v at room temperature (N-hexane and DCM) for around 30 min. The solution is applied with amorphous sulfate sodium and the next step is 2 mL of dried extracts. The extract close to sediment samples was washed with a silica gel column (Abbasi and Keshavarzi 2019; Sheikh Fakhradini et al. 2019). Following the EPA 418 protocol, the samples for TPH were analyzed. The samples have then been analyzed using a High Leistung Fluorescence Detector (HP-1046) Hewlett-Packard (HP) 1090 in the laboratory of Isfahan University of Technology using a RIGOL L-3000 High-Performance Liquid Chromatographer (HPLC, RIGOL Technologies, Inc., Beijing, China) equipped with a RIGOL L-3500 UV–vis detector (RIGOL Technologies, Inc., Beijing, China) and a Hewlett-Packard 1046 A fluorescence detector (Agilent Technologies, California, USA). A rotative vacuum evaporator concentrated the extracts to 1 ml. The PAHs analysis was based on 20 μl of each extract. At a rate of flux of 1 ml/min and temperature was set at 35 °C the mobile step was acetonitrile/water in gradient mode.

The water samples were immediately purified in the field by 0.45-μm Teflon filters. For PTEs analysis, the samples were acidified to pH < 2 with ultrapure nitric acid (HNO_3_) and kipped in dark polyethylene bottles at 4 ^◦^C prior to analysis of PTEs concentration by inductively coupled plasma-mass spectrometry (ICP-MS) at Zarazma Mineral Studies Company, Iran. Temperature, pH, and salinity of water samples were evaluated on-site using portable measuring devices (Eutech Instruments, PCD650).

### Quality assurance and quality control

Quality assurance and control (QA/QC) included the procedural blank, analytical duplicates and use of certified reference material (OREAS reference materials) for water and sediment samples. The recovery percentages of the investigated PTEs ranged from 80 to 110%, and the blank was below the detection limit. Concentrations of heavy metal(loid)s were recorded as mg/kg dry weight for sediments, and μg/L for water in this study.

Generally, the average recovery for TPH in sediment was approximately 88–95%. Reagent blanks, analytical duplicates/replicates, and analysis of the standard reference material (Dr. Ehrenstorfer GmbH Alkanes-Mix 10, and Sigma-Aldrich Co. LLC EPA 525 PAH Mix A and EPA 525 PAH Mix B) were processed.

### Data analysis

In order to estimate the pollution rate of sediments and water, this paper utilized different types of indices including enrichment factor (*EF*), geoaccumulation index (*I*_*geo*_), contamination factor (*Cf*), Nemerow pollution index (*NPI*), modified pollution index (*MPI*), and heavy metal toxic load (*HMTL*) to specify the pollution degree of PTEs in Hashilan wetland. In this study, potential ecological risk index (*RI*), modified ecological risk index (*MRI*), sediment quality guidelines (*SQGs*), toxic units (*TUs*), and toxic risk index (*TRI*) were applied to estimate the ecological risk in water and sediments of the wetland. To recognize the potential source of PTEs in sediments, Spearman correlation analysis, principal component analysis (PCA)/ absolute principal component scores (APCS) and positive matrix factorization (PMF) model were utilized. Also, partition coefficient (*K*_*p*_) was calculated to determine the interaction of PTEs between water and sediment phases. The details of the mentioned indices are described in the supporting information.

## Results and discussion

### Concentration of total petroleum hydrocarbons (TPH) in sediments

Total petroleum hydrocarbons (TPHs) values as the sum of aliphatic and aromatic hydrocarbons ranged from 4.2 to 76 mg/kg averaging 30.44 ± 28 mg/kg in sediment samples. The highest value of TPH was measured at the S2 site while S1 represented the lowest TPH concentration. The TPH values higher than 500 mg/kg in sediments indicate severely polluted sediments, while their contents below 10 mg/kg show no pollution (Akhbarizadeh et al. 2016; Kucuksezgin et al. 2012). Therefore, TPH concentrations in Hashilan sediment samples can be categorized as no polluted in site S1, and low polluted for the other collected sediment samples. Totally, the impact of human activities on the TPH concentrations in the Hashilan sediments was low.

TPH values in the sediments of Hashilan wetland are lower than those reported from Khark Island (80 to 618 mg/kg; Iran) (Akhbarizadeh et al. 2016), Yangtze estuary (50.05–428.50 mg/kg; China) (Li et al. 2019); Bohai Bay (6.3–535 mg/kg; China) (Zhou et al. 2014), Barnegat-Bay-Little Egg Harbor Estuary, USA (47–1003 mg/kg; USA) (Vane et al. 2008), but higher than those from the Bay of Bengal (1.8–40 mg/kg; India) (Venkatachalapathy et al. 2010) Bizerte lagoon (0.05–20 mg/kg; Tunisia) (Mzoughi et al. 2005); mangroves of the northern Persian Gulf (ND-1.7 mg/kg; Iran) (Mohebbi-Nozar et al. 2015) and Izmir Bay (0.43–7.8 mg/kg; Turkey) (Kucuksezgin et al. 2006).

### Concentration of heavy metal(loid)s in water and sediments

The PTEs values and statistical parameters in the collected sediments and surface water of the Hashilan wetland are summarized in Table 1. Regarding Levene’s test for equality of variances tests, all metal(loid)s concentrations in soil and sediment zones had values of greater than 0.05, proposing the assumption of equal variances is met (Table S2). Therefore, all soil and sediment samples were considered as a statistical population.

**Table.1:** Summary of statistical analysis of heavy metal(loid)s concentrations in surface water and sediments of the Hashilan wetland and comparison with water and sediment guidelines (WSA are from Taylor and McLennan (1985) and water standards are given from: https://www.fao.org/3/T0234E/T0234E01.htm)

Pollutants	Sediment (μg/g) *n* = 16									Water (μg/L) *n* = 6						Water Standards			
	Dl^a^	Min	Max	Mean	STD^b^	CV^c^	TEL	PEL	WSA^d^	Dl	Min	Max	Mean	STD	CV	EPA^e^	AWPL^f^	IWPL^g^	TRV^h^
Ni	1	17	136	80.94	40.16	0.5	18	36	18	0.1	< 0.1	< 0.1	-	-	-	-	20	-	87.71
Cr	1	17	210	111.13	62.05	0.56	37.3	90	42	1	0.67	1.37	0.98	0.34	0.35	100	5	8	117.3
Co	1	1.7	19.8	11.38	5.98	0.53	-	-	6.9	1	< 1	< 1	-	-	-	-	-	50	23
Sc	0.5	1.9	16.3	9.69	4.8	0.5	-	-	9.5	1	0.67	5.55	2.48	1.97	0.79	-	-	-	-
Mn	5	117	915	460.44	251.77	0.55	-	-	418	10	< 10	< 10	-	-	-	50*	-	200	120
Fe	100	9011	41,089	25,926.8	10,840.04	0.42	-	-	35,000	10	6.67	40	12.78	13.4	1.05	300**	300	5000	1000
Al	100	9805	63,889	39,383.7	17,473.26	0.44	-	-	71,000	10	10	30	18.33	7.53	0.41	50*	100	5000	87
Mo	0.1	0.07	7	1.2	1.59	1.33	-	-	1.8	0.1	0.85	4.35	2.74	1.3	0.47	-	73	-	370
Pb	1	2	21	10.56	5.4	0.51	35	91.3	25	0.1	0.67	4.4	2.9	1.35	0.47	15	7	200	9.05*
Zn	1	16	101	63.69	26.78	0.42	123	315	62	1	32.04	140.6	64.09	38.73	0.6	5000*	30	5000	212.5*
As	0.1	0.07	6.8	2.42	2.05	0.85	5.9	17	4.7	0.1	< 0.1	1.33	0.29	0.51	1.76	10	5	100	190
Cu	1	18	95	42.88	18.61	0.43	35.5	197	14	1	0.67	4.13	2.01	1.27	0.63	1300	4	200	23.8*
Cd	0.1	0.07	0.2	0.1	0.04	0.4	0.596	3.53	1.1	1	< 1	< 1	-	-	-	5	-		0.66

^a^Detection limit

^b^Standard deviation

^c^Coefficient of variation

^d^World soil average

^e^Environmental Protection Agency

^f^Aquatic Life Water Permissible Limits

^g^Irrigation Life Water Permissible Limits

^h^Toxicity Reference Value

Based on the results, Cu and Zn concentrations ranged from 18 to 95 mg/kg and 16 to 101 mg/kg, respectively. The elevated Cu contents were measured at stations S1 (95 mg/kg) and S2 (71 mg/kg). Also, the higher Zn concentrations were belong to sites S7 (101 mg/kg), S1 (92 mg/kg) and S2 (85 mg/kg). These stations have been located near the road, where agricultural machinery passes. The mean concentrations of these elements in the sediments of Hashilan wetland are higher than those reported from Shadegan wetland (Iran), Tijuana Estuary (California, USA), Nile delta (Egypt) and Sicily (Italy), but lower than those from Mangrove swamps (Hong Kong), Bellandur wetland (India), and Pearl River Estuary (China) (Table S3). The mean Cu value in the Hashilan wetland was about 3.06-fold higher than that in the world-soil average.

Nickel and Co concentrations varied from 17 to 136 mg/kg and 1.7 to 19.8 mg/kg, respectively. The higher Ni and Co values were observed at S10 (136 and 19.2 mg/kg, respectively) and S7 (134 and 19.8 mg/kg, respectively) sites. These stations are farmlands which are located near the road. The mean Ni and Co values (80.94 and 11.38 mg/kg, respectively) in Hashilan wetland were about 4.5-fold and 1.7-fold higher than those in the world-soil average.

The average Ni value in comparison with its mean contents in other investigated regions was high. Also, the mean Co concentration was lower than that from Anzali wetland (Iran) and close to those of Sundarban (India) and Shadegan (Iran) wetlands (Table S3).

Pb, Mn and Al contents varied from 2 to 21 mg/kg, 117 to 915 mg/kg, and 9805 to 63,889 mg/kg, respectively. The highest mean values of these elements belonged to the S7 station. The mean Pb, Mn and Al contents (10.56, 460.44, and 39,383.7 mg/kg, respectively) in the study area were below those of the world-soil average. However, the mean value of Pb in the sediments was higher than those from the Nile delta (Egypt), Mighan Wetland (Iran) and Sicily (Italia), but lower than other studies. Moreover, the mean Mn concentration in the study area illustrated a higher value than Shadegan wetland (Iran) and Sicily (Italia), but a lower value than Sundarban (India) and Sicily (Italia).

Arsenic concentration varied from 0.07 to 6.8 mg/kg with mean value of 2.42 ± 2.05 mg/kg. The elevated values of this element were observed at S9 (6.8 mg/kg) and S7 (5.9 mg/kg) stations. The average value of As in the study area was below those of the world-soil average and other investigated regions. Cr concentration varied from 17 to 210 mg/kg with mean value of 111.13 ± 62.05 mg/kg. The higher Cr contents were observed at S1 (210 mg/kg), S7 (195 mg/kg) and S9 (190 mg/kg) sites which have been located near the roads. Moreover, the average value of this element in Hashilan wetland in comparison with its mean contents in other investigated regions was high and also it was about 2.6-fold higher than that in the world-soil average.

Molybdenum concentration ranged from 0.07 to 1 mg/kg in all sampling sites except S3 station with 7 mg/kg. The maximum Mo value in the Hashilan wetland was about 3.8-fold higher than that in the world-soil average. Also, the range of Cd concentration was small, varying from 0.07 to 0.2 mg/kg. The mean Cd content (0.1 mg/kg) in the study area was below that of the world-soil average. However, its mean value in the sediments was higher than those from Plateau lake wetland (China), Hengshuihu Wetland (China) and Nile delta (Egypt), but lower than other studies.

Totally, the average values of the investigated elements decrease as follows (mg kg^−1^): Al (39,383.7) > Fe (25,926.8) > Mn (460.4) > Cr (111.12) > Ni (80.94) > Zn (63.7) > Cu (42.9) > Co (11.4) > Pb (9.7) > As (2.4) > Mo (1.2) > Cd (0.1). The coefficient of variation (C.V) is defined as the standard deviation to the mean ratio, which could reflect the degree of humankind effects (Guan et al. 2019; Wu et al. 2020). The coefficients of variation of As, Cr, Mn, Co, Ni, Pb, Sc, Al, Cu, Fe, Zn, and Cd were 85%, 56%, 55%, 52%, 50%, 50%,50%, 50%, 44%, 43%, 42%, 42%, and 40%, respectively. The variation degree of these elements was between 0.1 and 1, indicating moderate variability (Jin et al. 2019). While, the variation degree of Mo was greater than 1, which is categorized as strong (Jin et al. 2019). The high coefficient of variation of Mo proposed that human activities could affect its value in the sediment of the wetland.

The elements of Ni, Mn, Co, and Cd were not detectable in the surface water of Hashilan wetland, which caused the portions of these heavy metals to be negligible for water pollution. Cr and Fe elements were detectable at two or three sites. The elements of Cu and Mo contents ranged from 0.7 to 4.13 µg/L, and 0.85 to 4.35 µg/L, respectively. The higher values of Cu and Mo were observed at S3, S8, and S12 sites where the pollutants values increase due to stagnant water. Phosphate fertilizers, pesticides or fungicides contain Cu and Mo elements which could be the source of these elements at S8 (the outlet of agricultural wastewater into the wetland) (Li et al. 2020). Also, wear dust from brake linings and tires of vehicles can be considered the source of these elements at S3 (near the road) (Lin et al. 2015). Zinc value varied from 32.04 to 59.13 µg/L in all sampling sites except the S8 station with 140.58 µg/L where agricultural runoff entered the wetland. Lead concentration ranged from 0.67 to 4.4 µg/L with a mean value of 2.9 µg/L. The higher Pb contents were observed at S2 (4.4 µg/L), S8 (3.79 µg/L) and S1 (3.64 µg/L) sites. Aluminum and Sc concentrations ranged from 10 to 30 µg/L and 0.67 to 5.55 µg/L, respectively. The highest contents of these elements were measured at stations S12 and S8, respectively. Also, As values were only detected at S8 and S12 stations. Totally, the average values of the investigated elements decrease as follows (µg l^−1^): Zn (64.09) > Al (18.33) > Fe (12.78) > Pb (2.9) > Mo (2.74) > Sc (2.48) > Cu (2.01) > Cr (0.98) > As (0.29). The concentrations of the investigated metal(loid)s were below the recommended drinking water standards by EPA (USEPA 2012) and WHO (2017).

### Assessment of PTEs contamination

#### Sediment quality assessment

##### Geoaccumulation index (Igeo)

The calculated I_geo_ values for PTEs in sediments of the Hashilan wetland are presented in Fig. S1. Lead, Cd, As, Fe, and Al in the Hashilan sediments were classified as “unpolluted” status as a result of the I_geo_ index lower than Zero, while the I_geo_ values of Sc, Co, Mn, and Zn fluctuated from “unpolluted” to “unpolluted to moderately polluted” status. Comparatively, the *I*_*geo*_ values for Cu and Ni varied from -0.22 to 2.18 and -0.67 to 2.33, respectively, suggesting “no contamination” to “moderate to strong contamination.” Chromium in sediments was classified as no pollution to moderate pollution. The I_geo_ values for Mo showed an “unpolluted” status at all sampling sites except S3, which presented moderate pollution. Totally, the average I_geo_ values of the investigated metal(loid)s were as follows: Cd (-4.10), As (-2.64), Pb (-2.06), Mo (-1.99), Al (-1.63), Fe (-1.16), Sc (-0.81), Zn (-0.72), Mn (-0.69), Co (-0.16), Cr (0.48), Cu (0.92), and Ni (1.34), proposing the tested metal(loid)s except Ni, Cr, and Cu indicated no pollution in general. Also, the average contents of I_geo_ for Ni indicated moderate pollution, while those of Cr and Cu demonstrated uncontaminated to moderately contaminated conditions.

##### Enrichment factor (EF)

The calculated EF values for PTEs in sediments of the Hashilan wetland are presented in Fig. S2 (SI 2). The EFs values calculated revealed that Al, As, Cd, Co, Mn, Pb, Sc, and Zn were minimal enriched at all sampling sites, which indicate that the mentioned elements were mainly originated from crustal materials or natural weathering. Also, the sediments were moderately enriched with Ni at all stations. Enrichment factors of Cr were also higher or much closer to 2 in all the sampling sites, indicating the influence of anthropogenic pollution in sediments. According to studies conducted in Iran, the chromium and nickel concentrations in the soil of Iran country (especially near the Zagros mountains) are higher than those of the global average of soil and upper crust (Abbasi et al. 2018, 2019). Despite moderate EFs of Ni and Cr, these elements showed low and uniform EF ranges which illustrate a geogenic source. Ophiolite sequences of the Zagros fold-and-thrust belt are associated with high levels of toxic trace elements,

particularly nickel, chromium, and cobalt. Therefore, soils formed over them are polluted by the mentioned metals were controlled by weathering processes of parent materials (Allahyari et al. 2010; Namaghi et al. 2011).

Mo showed significant and moderate enrichments at S3 and (S5 and S6) stations, respectively, while other stations were relatively unpolluted. Copper (Cu) revealed significant and moderate enrichments at (S5, S6) and (S9, S10 and S13) stations, respectively, while other stations indicated deficiency to minor enrichments. Therefore, the anthropogenic sources are suggested for Cu, and Mo at some stations. Totally, the mean EFs of trace elements were ranked as follows: Ni (4.44) > Cu (3.8) > Cr (2.5) > Co (1.6) > Mn (1.14) > Zn (1.08) > Mo (1.04) > Fe (0.81) > Al (0.57) > As (0.45) > Pb (0.44) > Cd (0.13), suggesting the tested metal(loid)s except Ni, Cu, and Cr illustrated minimum enrichment in general. While, the average contents of EFs for Ni, Cu, and Cr indicated moderate enrichment.

##### Contamination factor (CF)

The calculated CF values for PTEs in sediments of the Hashilan wetland are presented in Fig. S3. Contamination factor (CF) values calculated showed that all the sediment samples have been low contaminated by Al, Cd, Pb, and Mo elements. Also, As revealed minor contamination for all sampling sites except S7 and S9 which were moderately polluted. Zinc and Co showed a moderate degree of pollution at most stations. Chromium illustrated low, moderate, and considerable contaminations at (S5, S6, S8), (S3, S4, S11, S15, S16) and other stations, respectively. Moreover, serious contamination of Ni was observed at S1, S7, S9, and S10 stations, also half of the stations were considerably polluted by this heavy metal. Copper showed serious contamination at the S1 station, while moderate to considerable contaminations of Cu were observed for other sampling sites. Manganese, Sc, and Fe showed low to moderate contaminations in the Hashilan sediments. The mean CFs of trace elements were ordered as follows: Ni (4.5) > Cu (3.1) > Cr (2.64) > Co (1.65) > Mn (1.1) > Zn (1.03) > Sc (1.02) > Fe (0.74) > Mo (0.67) > Al (0.55) > As (0.51) > Pb (0.42) > Cd (0.09), suggesting the investigated metal(loid)s except Ni, Cu, Cr, Co, Mn, Zn, and Sc demonstrated low contamination in general. While, the mean contents of CFs for (Ni, Cu), and (Cr, Co, Mn, Zn, Sc) showed considerable and moderate pollutions, respectively.

##### Modified pollution index (MPI) and Nemerow pollution index (NPI)

According to Table S4 and Fig. S4, *MPI* (modified pollution index) revealed that S5 and S6 sites have been severely polluted, while other sampling stations except S14 were moderately heavily polluted. Also, the site of S14 indicated moderate pollution.

*NPI* (pollution index) indicated S1, S2, S4, S7, S9, S10, S12, S13, and S14 sites were heavily polluted, and the rest sampling stations have been severe to moderately polluted. It seems that NPI has overestimated the pollution degree at the sampling sites compared to MPI, which is computed by the enrichment factor (Fig. S4).

#### Water quality assessment

##### Heavy metal toxicity load (HMTL)

To evaluate the HMTL, the potentially toxic metal(loid)s including As, Cu, Pb, Zn, Cr, and Al were chosen from the ATSDR substance priority list (ATSDR 2017). Also, different guidelines including aquatic life water permissible limits (AWPL) (CCME 2007), toxicity reference values (TRVs) (WSRC 1999) and recommended drinking water standards by the environmental protection agency (EPA) (USEPA 2012) were used to assess different permissible toxicity loads. In the study area, HMTL ranged from 55.35 to 153.56 mg/L with an average of 78.48 mg/L. The HTML calculated was less than the permissible toxicity loads prepared by EPA and TRV guidelines (5774. 8 and 966.5 mg/L, respectively), suggesting low pollution of PTEs at all sampling sites, while HTML of the S8 station (153.56 mg/L) was above the permissible toxicity load prepared by AWPL guideline (119.1 mg/L) (Table 2). However, the pollution loads of Cu at S3, Cr at S1, S2, S8 and Zn at all monitored stations were above their corresponding permissible toxicity loads prepared by AWPL guideline, proposing the reduction of Zn, Cr, and Cu contents of the Hashilan water. However, this study has evaluated the water quality with respect to some heavy metals, while other organic and inorganic pollutants listed in the ATSDR substance priority list could be considered to obtain a better insight into the water quality of the study area.

**Table.2:** Heavy metal toxicity load of the surface water based on relative toxicity level of heavy metal(loid)s

Toxicity of heavy metals (mg/L)							
**S**ampling sites	Cu	Pb	Zn	As	Cr	Al	Heavy metal toxicity load (HTML) (mg/L)
S1	1.30	5.57	53.99	0.11	1.13	6.85	68.96
S2	0.85	6.74	53.03	0.11	1.10	6.85	68.67
S3	3.32	1.03	43.33	0.11	0.60	13.70	62.09
S5	0.54	4.19	43.14	0.11	0.60	13.70	62.28
S8	2.25	5.80	128.35	2.23	1.22	13.70	153.56
S12	1.46	3.28	29.25	0.22	0.60	20.55	55.35
Total	9.72	26.61	351.09	2.89	5.24	75.35	
Hazard Intensity Score^*^	805	1531	913	1676	893	685	
Permissible toxicity load (EPA)^a^	1046.50	22.97	4565.00	16.76	89.30	34.25	5774.80
Permissible toxicity load (TRV)^b^	18.58	13.86	194.01	318.44	362.02	59.60	966.50
Permissible toxicity load (AWPL)^c^	3.22	10.72	27.39	8.38	0.89	68.50	119.10

*ATSDR 2017

^a^Environmental Protection Agency

^b^Toxicity Reference Value

^c^Aquatic Life Water Permissible Limits

Ecological risk assessment for heavy metal(loid)s in sediments

##### Nemerow pollution index (NPI)

To evaluate the Nemerow pollution index, reference values were selected from AWPL, TRVs and EPA guidelines to provide more accurate information about the pollution rate in the surface water.

The single factor index values of Zn at all stations, and Cr at S1, S2, S8 sites calculated by AWPL as a reference value exceeded 1 which caused NPI > 1 at all stations, representing serious pollution of metal(loid)s at all monitoring sites (Fig. S5). The highest NPI value was 3.41, which was detected at the S8 station. However, Zn and Cr were considered to be the significant factors for NPI > 1. In comparison, the single factor index values of the investigated PTEs calculated by EPA and TRV guidelines as the reference values were below 1 which caused NPI < 1 at all sampling sites, showing no pollution threat at all stations (Fig. S5). The results indicated the pollution rate of water could be dependent on the selected reference values. However, agricultural activities have affected the aquatic environment of the Hashilan wetland. Therefore, to protect the wetland environment, the pollution rate is required to be controlled.

##### Mean-PEL-quotient

The comparison between sediment quality guidelines and metal concentrations in sediment is applied to evaluate the contamination effects on the biota (Maanan et al. 2015). Copper values at most stations except S8 were above the respective TEL content; even though, its concentrations were lower than the respective PEL value. Nickel concentrations were above the respective PEL value at all sampling sites except S5, S6, and S8 stations which could create potential adverse biological consequences at these sites. In the case of Cr, its contents exceeded the respective PEL value at S1, S7, and S9 sites which could cause potential adverse ecological risk at the mentioned stations, while its maximum values at S5, S6, and S8 stations were lower than the respective TEL content. Moreover, the As, Cd, Pb, and Zn values in all sediment samples were observed to be below the respective TEL contents, proposing that these elements in Hashilan wetland would not be associated with adverse ecological risk.

However, the mean PEL quotients were between 0.11 and 1.5 at all sampling sites, representing low to moderate ecological risk for an aquatic organism, with a toxicity occurrence of between 10 and 25%.

##### RI and MRI

The highest and lowest values of Er_i_ in sediments of the Hashilan wetland were belong to Cu and As elements, respectively. The rest of the heavy metals were observed in between the two extremes. Computed Er_i_ with enrichment factor indicated a moderate risk of Cu at the only S5 site, while other elements exhibited low risk at all locations. Computed Er_i_ with contamination factor in comparison with Er_i_ with enrichment factor showed that the ecological risk of Cu has decreased to low risk at S5 station. Furthermore, all the heavy metal(loid)s presented low risk at all sites.

MRI (modified ecological risk index), which is calculated by enrichment factor, exhibited low ecological risk at all sampling stations. Also, according to RI (potential ecological risk index), which is calculated by contamination factor, all stations caused low ecological risk for ecosystem (Fig. S6 and Table S4).

##### ΣTUs and TRI

The ΣTUs* and* TRI values for Hashilan sampling sites are demonstrated in Fig. S7 and Fig. S8, respectively. The ΣTUs values for all sediment samples varied from 0.8 to 5.52. Based on the ΣTUs results, five sampling sites demonstrated moderate toxicity risk due to ΣTUs values greater than 4.0, while other sampling sites indicated low toxicity to the ecosystem. According to the contributing mean ratios of the metal/loids toxic units (TUs) in all sediment samples to ΣTUs, Ni (54.72 ± 5.20%), Cr (19.16 ± 3.73%), and Cu (13.66 ± 6.46%) presented higher potential toxicity compared to the Zn (7.16 ± 0.95%), Pb (2.85 ± 0.89%) As (1.5 ± 1.07%), and Cd (0.97 ± 0.74%). Also, TRI values ranged from 2.45 to 15.95, with the highest value at S1 and the lowest at S6, which was consistent with the ΣTUs contents. Compared with the ΣTU contents, 2 stations (S1 and S7) indicated considerable toxicity risk with the TRI values greater than 15. Also, seven stations exhibited moderate toxicity risk with the TRI values varying from 10 to 15. Meanwhile, low toxic risk was measured at 4 stations with TRI values varying from 5 to 10. Three stations also showed no toxic risk with TRI values lower than 5. However, the areas with higher potential risk were mainly focused in the vicinity of roads.

The mean contribution ratios of each metal(loid) to the TRI contents were 54.78 ± 6.43% for Ni, 19.44 ± 8.27% for Cu, 15.24 ± 3.25% for Cr, 4.23 ± 0.56% for Zn, 2.7 ± 0.85% for Pb, 2.18 ± 1.55% for As, and 1.44 ± 1.03% for Cd. Moreover, compared with the ratio of Cu, As, and Cd in the ΣTUs, the contributing ratio of the mentioned elements was increased according to the TRI, proposing higher potential toxicity of the mentioned elements. Cu and Zn showed a lower toxicity contribution in the TRI compared to the ΣTUs. In addition, according to ΣTU and TRI contents, the highest contribution ratio has belonged to the Ni element.

According to the results, the ΣTUs method demonstrated the lower potential toxicity risk compared to the TRI method because the TRI index applied the TEL and PEL values, providing more reference contents compared to the ΣTUs, which only consider high PEL values for toxicity risk estimation (Ji et al. 2019a).

It is worthy to note that several methods of risk evaluation utilize different toxicity parameters or values of PTEs, which cause different index contents or risk levels. Among the MRI, RI, TRI, Mean-PEL-quotient, and TUs methods, TRI equation better presented the toxicity risk of heavy metals regarding the results of MPI and PI indices. However, the risk level of Mean-PEL-quotient is higher than that of the MRI and RI methods. Totally, TRI index is more suitable for the PTEs risk assessment in the sediments of Hashilan wetland.

#### Ecological risk assessment for heavy metals in water

Water quality guidelines of heavy metals for the protection of aquatic Life and agriculture proposed by the Canadian Council of Ministers of the Environment (CCME 2007) were used as the water quality reference values due to the agricultural activities and existence of unique species in the Hashilan wetland. Also, another reference value called TRVs (WSRC 1999) was used to evaluate the ecological risk of Hashilan water.

The ecological risk index calculated by three reference values including aquatic life water permissible limits (AWPL), irrigation life water permissible limits (IWPL) and aquatic toxicity reference values (TRVs) ranged from 5.8 to 16.3 with a mean of 9.25, 0.27 to 0.67 with mean of 0.41, and 1.4 to 3.4 with mean of 2.36, respectively (Fig. S9). The RI results indicated a low ecological risk for all sampling sites. With respect to the single metal risk index, the ecological risk of all the investigated PTEs in surface water was low.

### Source identification of heavy metals in the sediments

#### Correlation analysis

The Spearman correlation coefficients among trace metal/metalloids and those between metals and physiochemical parameters in the sediment system are illustrated in Table S5. These relationships could be applied to determine PTEs sources (Ji et al. 2019b; Xiao et al. 2019). Also, the mobility and bioavailability of heavy metals in sediments could be affected by sediment properties like organic matter, particle composition, EC and pH (Gao et al. 2016).

Particle size can modify the distribution and adsorption behavior, erosion and remobilization of heavy metals (Huang et al. 2020). Among different particles, fine grains tend to absorb more heavy metals by providing a high specific surface area and binding sites (Gao et al. 2016; Hu et al. 2018). According to the correlation coefficients, silt particles were weakly correlated with the studied elements, while clay particles illustrated positive correlation with Ni, Al, Fe, Sc (*p* < 0.01, *r* = 0.7); Co (*p* < 0.01, *r* = 0.6); Cr, Pb, Zn (*p* < 0.05, *r* = 0.5) which indicate the mentioned metals tend to accompany with the proportion of clay. However, only a negative correlation was found between clay particles and Mo (*p* < 0.05, *r* = -0.5). Also, sand particles were negatively correlated with the investigated PTEs except for Mo, suggesting these heavy metal(loid)s could be easily released from the sand grains (Yavar Ashayeri and Keshavarzi 2019; Khalil and El-Gharabawy 2016).

Organic matter as a significant sink tends to create complexes with metals via providing certain ligands (Gao et al. 2016; Zhu et al. 2017). However, OM was negatively correlated with Co, Fe (*p* < 0.01; *r* = -0.8); As, Ni, Al, Cr, Sc (*p* < 0.01; *r* = -0.7), and Pb, Zn (*p* < 0.01; *r* = -0.6), indicating distribution and adsorption of the investigated elements has been less affected by organic matter compared to the other influential factors and adsorbents. Organic matter (OM) showed a significant positive correlation with sand (*p* < 0.05; *r* = 0.5). The higher contents of organic matter were found at the stations in which agricultural wastewater enters the wetland because not only agricultural runoff contains high values of organic matter (Zhu et al. 2017) but also dense vegetation is observed at these situations. At the mentioned stations, as the runoff enters the wetland, the flow velocity of agricultural runoff decreases which causes the coarse particles to be firstly deposited in these positions. Therefore, the association of sand particles and organic matter is observed in these stations which causes a strong correlation between them. Molybdenum also had a positive correlation with OM and sand particles. Such correlations could be related to the same origin or transport path for them (Liu et al. 2019). Also, this heavy metal indicated a weakly positive correlation with Cu, while no correlation was found between Mo and other elements, suggesting their concentrations may be controlled via different sources which cause the distributional difference between them (Suresh et al. 2012; Yuanan et al. 2020). A significant correlation was not found among cation exchange capacity (CEC) and other elements and parameters, and only a weakly positive correlation was observed between CEC and clay particles. Most studies emphasize the CEC value depends on clay particles and organic matter, while the elemental composition of clay particles must be mentioned (Malcolm and Kennedy 1970). Also, CEC values of greater particles size could be higher than clay particles, depending on parent material, age and degree of weathering, type of clay minerals, climatic factors, and physical and chemical dispersion (Malcolm and Kennedy 1970). Therefore, in order to understand the controlling factors of CEC parameters, the mineralogy of particles, type of organic matter and other effective parameters should be studied.

Spatial distribution patterns, transfer and mobility of PTEs could be affected by Fe/Mn oxides (Ji et al. 2019b). However, Fe and Mn were significantly correlated with the studies elements except As, Cd and Mo in sediments. Nickel, Pb, Zn, Cr, Co, As, and Sc revealed a significant positive correlation with each other (*r* > 0.6). Copper also showed a positive correlation with As, while this heavy metal was significantly and positively correlated with the mentioned elements, which indicate the same source probably and/or similar geochemical behaviors for the mentioned PTEs (Suresh et al. 2012; Xiao et al. 2019; Zhu et al. 2017). However, Sc is an immobile metal that originated from lithologic sources (Li et al. 2020), showing the parent rock has impressed the contents of metal(loid)s associated with Sc. Cadmium illustrated no significant relationship with metal(loid)s and parameters, and only a weakly positive correlation was observed among this heavy metal and Cu, Pb, Zn, Ni, and Cr (*P* > 0.05; *r* = 0.3). These results may be due to the similar values and limited fluctuations of Cd concentrations at most sampling sites.

The mobility of elements could also be controlled by sediment pH (Fei et al. 2019; Ustaoğlu and Islam 2020). In the study area, sediment’s pH was weakly correlated with the investigated metals as a result of the limited variability and alkaline status of pH values.

#### Principal component analysis (PCA)

Principal component analysis was firstly employed for identifying the potential contamination sources of PTEs. The measured KMO value and significance level of Bartlett's Sphericity test were 0.7 and 0 (< 0.05), respectively, indicating the suitability of data for performing PCA analysis. Kaiser's rule was utilized to specify the components extracted from the variables. The factor loadings > 0.75, 0.75–0.5 and 0.5–0.3 are defined as “strong”, “moderate” and “weak”, respectively (Zeng et al. 2019; Zhang et al. 2020). Table 3 illustrated the factor loadings of the studied PTEs at the extracted components.

**Table.3:** The rotated factor pattern derived from PCA for the contents of heavy metal(loid)s in the sediments of Hashilan wetland

Principal component		
	**1**	**2**
**Mo**	-0.28	**0.85**
**Cu**	**0.57**	**0.54**
**Pb**	**0.90**	0.20
**Zn**	**0.93**	0.28
**Ni**	**0.97**	0.06
**Co**	**0.98**	0.08
**Mn**	**0.90**	0.04
**As**	**0.79**	0.07
**Cd**	0.32	0.33
**Cr**	**0.95**	0.24
**Al**	**0.98**	0.09
**Sc**	**0.97**	0.07
**Fe**	**0.98**	0.01
**Eigenvalue**	9.40	1.18
**% of variance**	71.40	10.20

Bold figures indicating high positive correlation of initial variables to rotated fact

The PCA performance identified two varimax rotated factors with eigenvalues greater than 1 and accounting for 81.62% of the total variance. The first principal component (PC1) accounted for 69.75% of the total contribution with high Pb, Zn, Ni, Co, As, Mn, Cr, Al Sc, Fe loadings and moderate Cu and weak Cd weights, which is in agreement with the results of the correlation analysis.

Based on the EF assessment, the mentioned elements except Ni and Cr exhibited depletion or minor enrichment in the sediments. Since the Ni and Cr contents in soils mainly depend on their concentrations in parent rocks, and human inputs of Ni and Cr from manure, limestone, and fertilizers are generally less than their background values in soils (Lv 2019), these heavy metals have been moderately enriched due to the geogenic source. Totally, a natural source (parent rock materials) could be attributed to this component due to the presence of lithogenic elements such as Fe, Sc, Mn, and Al in PC1 (Qiutong and Mingkui 2017; Zhu et al. 2019).

PC2, with 10.25% of the total variance and 1.18 of the Eigen value, indicated a high weight of Mo, moderate Cu loading and weak Cd weight. According to the EF values, Mo and Cu demonstrated middle to extreme enrichments at some stations. Also, wide and skewness EF values were observed for Mo and Cu. Phosphate fertilizers, which are used in croplands, contain some metals such as Cu, and Mo (Azzi et al. 2017; Gupta et al. 2014). Also, Cu is extensively utilized as a significant metal for pesticides or fungicides (Li et al. 2020; Liang et al. 2017). On the other hand, wear dust from brake linings and tires of vehicles and agricultural machinery can be a source for the release of these elements into the environment (Lin et al. 2015). Thus, this factor is related to both natural sources and agricultural activities.

Copper on two principal components showed moderate loading, which suggested two sources of principal components (natural, and mixed sources) could be considered for this metal. Moreover, Cd metal showed ambiguous attribution and weak load on two principal components. The correlation analysis illustrated this element has no significant correlation with other elements. Also, the Cd concentrations were evaluated low and uniform, suggesting this heavy metal was derived from lithogenic origins.

Then, the percent contribution of each component was assessed by multiple linear regression via performing a stepwise procedure. Two principal component scores including natural and mixed sources were regressed against the investigated metal(loid)s. The obtained equation is presented as:

1 $$\mathrm{Z}=0.981\mathrm{PC}1+0.057\mathrm{PC}2 ({\mathrm{R}}^{2}=0.96)$$

Then, the regression coefficients detected were applied to estimate the portion of each source, presented by principal components. Based on the results, the contribution of natural sources was 94.51% of the total portions and the mixed sources containing geological origin and agricultural activities (fertilizers and agricultural equipment) contributed 5.5% of the total shares.

Although the contribution rates of two sources were specified, the PTEs portions in each source could not be achieved due to negative values detected in component scores derived from the APCS-MLR model (Dong et al. 2019).

#### Positive matrix factorization model

To verify the extracted sources of PCA analysis, the PMF model was employed not only to recognize the sources of heavy metals but also to quantify the metal(loid)s contributions in sediments (Dong et al. 2019; Yuanan et al. 2020).

**Fig. 2:**
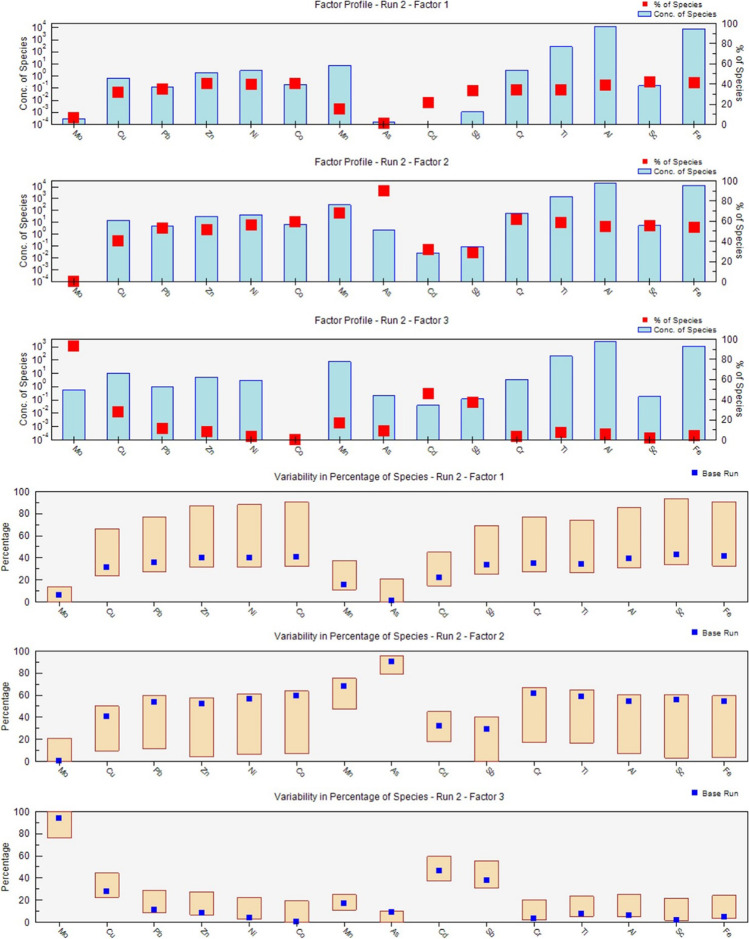
Results of PMF source apportionment modeling for heavy metal(loid)s in the sediments of Hashilan wetland

The PMF model run was 20 times and the seed number was randomly selected. To detect the minimum Q value and the optimal number of origins, two to five factors were tested. The lowest and stable amount of Q was achieved when the factors extracted were three (Fig. 2). Also, the signal-to-noise ratios (S/N) of the investigated metal(loid)s were higher than 1, indicating the data qualities are strong (Mao et al. 2020; Wu et al. 2020).

Also, the scaled residuals of sediment samples illustrated a normal distribution between -3.0 and 3.0. Moreover, Q_robust_ /Q_true_ ratio was 0.96 and R^2^ between the predicted and measured values of all metal(loid)s was greater than 0.6, proposing the factors extracted by the PMF model are reliable (Fig. 2). The validity of PMF results was estimated via the performance of 80 bootstrap runs with a minimum *R*^2^ value of 0.6.

**Fig. 3:**
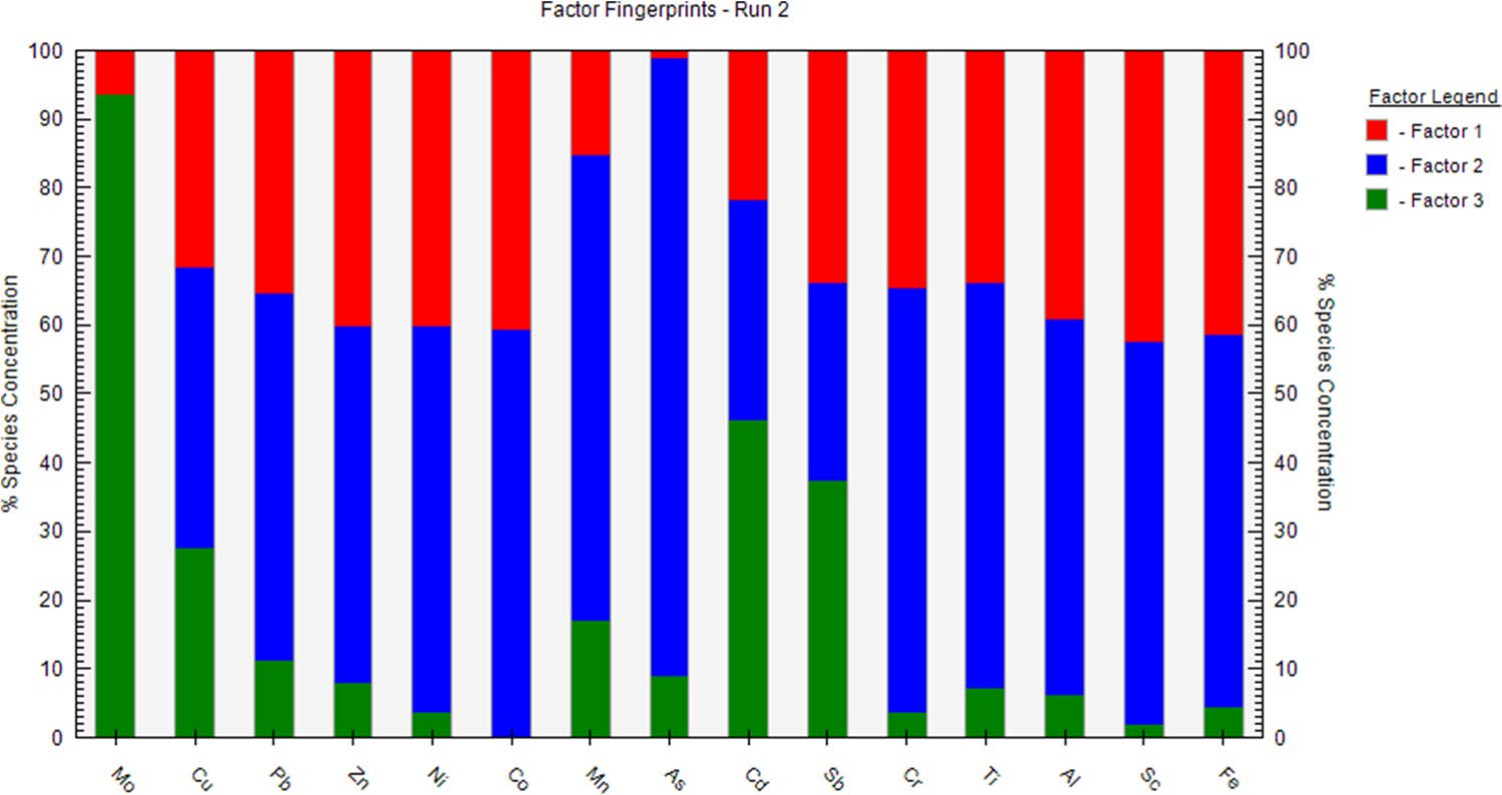
Contribution of the heavy metal(loid)s according to factor profiles obtained by PMF model

According to Fig. 3, Factor 1 (F1) was dominated by Sc (42%), Fe (41%), Co (41%), Ni (40%), Zn (40%), Al (39%), Pb (36%), Cr (35%), Cu (32%), followed by Mn (15%), Cd (12%), Mo (6%) and As (1%). The metal(loid)s in Factor 1 were again categorized into a group (Factor 2) for PMF, which was presented by As (80%), Mn (68%), Ni (67%), Cr (61%), Co (59%), Sc (56%), Al = Fe (55%), Pb = Zn (52%), Cu (41%), and Cd (32%). As mentioned, As and Mn elements showed natural sources based on the EF and I_geo_ equations. However, higher percentages of these elements in F2 compared to the F1 may be related to a high CV of their concentrations. According to various researches, the concentrations of Fe, Al, Sc, Mn, Cr, and Ni are controlled by parent materials (Dong et al. 2019; Liang et al. 2017; Lu et al. 2020). These two factors, similar to factor 1 of the PCA model, propose the mentioned metal(loid)s were released from natural weathering of rock materials (Harikrishnan et al. 2017).

Factor 3 indicated high weights of Mo (94%), Cd (46%), and Cu (27%), while its contributions to Mn (17%), Pb (12%), As (9%), Zn (8%), Al (6%), Cr = Fe (4%), Ni (3%), and Sc (2%) were relatively low. As mentioned, EF values of Mo and Cu were middle to extreme at some stations. Also, Mo and Cu are found in fertilizers, manures and other agricultural activities (Azzi et al. 2017). Thus, this factor would describe the same pollution source (mixed natural and agricultural origins) as PC2 of PCA analysis.

Similar to PCA analysis, which indicated analogous and weak weights of Cd in two PCs, the cadmium contributions between F3 and (F1 and F2) of the PMF model were almost similar. However, the *EF* and *I*_*geo*_ of Cd illustrated the weathering of parent rock has controlled its concentrations in soil and sediment of Hashilan wetland. Copper also showed a similar load on two principal components in the PCA analysis, while Cu in the PMF model accounted for 27% of factor 3 and 73% of F1 and F2, proposing the natural sources include a larger contribution of Cu compared to the agricultural activities.

Due to the different algorithms used by PCA and PMF models, the results of source contributions illustrate differences. Thus, the comparison of models helps to obtain more detailed information of metal(loid)s sources.

### Sediment–water distributions

Some agents such as pH, metal contents in soluble and solid phases, the values of metal complexing agents, and the physio-chemical properties of water and sediments affect the partition coefficient of elements in the water–sediment system (Allison et al. 2005). High contents of K_p_ (log K_p_ > 2.9) indicate that PTEs prefer to be available in the sediment phase; whereas, low contents (log K_p_ < 2.9) show that the solution has preferentially preserved the dominant portion of metal(loid)s (Jung et al. 2005).

Among the PTEs, Ni presented the highest K_p_ with 6.25 L/kg (S1 site); whereas, the lowest K_p_ content belonged to the Cd with 2 L/kg (S5 site). The mean values of K_p_ decreased as (L/kg): Ni (5.92) > Cr (4.92) > Mn (4.67) > Cu (4.43) > Co (4.05) > Pb (3.54) > As (3.53) > Zn (2.96) > Mo (2.76) > Cd (2.20) (Table S6).

Nickel, Cr, Mn, Cu, Co, and Pb indicated higher contents of K_p_ (log K_p_ > 2.9) relative to other elements, proposing their lower solubility in water and higher affinity to be adsorbed to sediment samples. The relatively higher K_p_ (log K_p_ > 2.9) of As and Zn were recognized at most stations, suggesting these elements prefer to be adsorbed in solid-phase compared to soluble phase. However, As and Zn were present in the water phase at (S8, S12) and (S5, S8) stations, respectively.

In contrast, relatively lower K_p_ of Cd (log K_p_ < 2.9) proposed the lower affinity of solid phase to adsorb this element. The distribution coefficient of Mo indicated that this element is more present in soluble phase relative to solid-phase except for S3, and S8 sites due to high mobility of Mo at natural to alkaline conditions (Smedley and Kinniburgh 2017).

In the Hashilan wetland, the median log K_p_ contents for As, Co, Cr, Cu, Mo, and Ni were, 3.7 L/kg,4.2 L/kg, 5.13 L/kg, 4.6 L/kg, 2.7 L/kg, and 6.02 L/kg respectively, which presented the higher values relative to the respective median contents for the EPA (Allison et al. 2005).

#### Correlation of phase partition with influential factors

Clay particles tend to adsorb heavy metals due to the negative charge and high surface area (Zhao et al. 2013). In this study, clay particles indicated the weakly positive trend with partition coefficients of Cu, Zn, Ni, Co, Cd, Fe, Al (*r* < 0.5) and a significant opposite trend with Mo (*r* = -0.8). Also, silt particles showed a negative correlation with partition coefficients of Fe, Sc (*r* = -0.9), and Cu, Zn, Ni, Co, Cr, As, Al (*r* = -0.7) (Table S7). However, smaller grains are favorable to desorb or adsorb the metals (Feng et al. 2017). The positive correlation of OM with partition coefficients of Pb and Mo (r = 0.4) showed that these particles create bonds to prevent Pb and Mo liberation. However, a negative correlation of OM with partition coefficients of Cu, Ni, Co, As, Cd, Fe, Sc, Al, and Mn (*r* = (-0.3)-(-0.7)) was observed. In the investigated stations, pH values showed opposite trend with Mo and As (*r* = -0.9 and -0.5, respectively), while this parameter did not affect the distribution coefficients of the other mentioned elements (Table S7). However, although the range of pH fluctuations is small, varying from 6.50 to 6.88, this factor could influence the Mo and As desorption from sediments. In this study, the effect of other influential factors like Fe–Mn oxides concentrations in sediments, and the sorbents nature and their values in water on the metal partitioning must be examined.

### Agricultural nutrient pollutants and eutrophication

In wetland ecosystems, the fate of nutrients (N and P) is determined by a combination of natural processes and human activities such as agriculture (Uwimana et al. 2018). Due to the unreasonable and excessive application of pesticides and fertilizers, and agricultural non-point source contamination has led to severe eutrophication in downstream wetlands (Yu et al. 2018). The increase in nitrate and phosphate contamination in surface water and groundwater of the study area is associated with intensive agriculture activities that are lead to an increased load of nutrient pollutants through the use of nitrogen and phosphate fertilizers. On the other hand, high N and P concentrations are the main cause of the eutrophication of wetland water. Not only do these impact the ecological character of wetlands, but they also have effects on human health and the quality of drinking water supplied from wetlands (Ramsar 2014). Therefore, in this study, the contamination of N and P compounds as nutrient pollutants in the water is compared with drinking and agricultural water standards. As shown in the Table S8, the total nitrogen (TN) concentration in water samples was varied between 8.63 mg/l and 48.16 mg/l. Exceptionally high TN concentration were observed at S8 site which could be related to agricultural activities which has likely mostly increased the concentration of TN in the station due to the use of chemical fertilizers.

Nitrate (NO_3_^−^) is typically the dominant form of N in natural waters and polluted water. Nitrate and other forms of nitrogen in an aquatic ecosystem can originate from natural sources, but when N values are elevated, the sources are commonly related to anthropogenic sources (MPCA 2013). In Fig. S10 and Table S8, the values of nitrate (NO_3_^−^) in water sampling stations are observed. NO_3_^−^ values in all stations (except S8) were lower than the value reported by FAO (1973). In the S8 station, the NO_3_^−^ concentration was 37.08 mg/l, due to water samples being taken near the farms. However, the mean NO_3_^−^ concentration was relatively higher in wetland water (10.39 mg/l), which showed the use of massive anthropogenic fertilizers has caused an additional NO_3_^−^ input enters wetland water, which can pose a threat to increase the risk of water eutrophication. This is of particular importance in wetland ecosystems with a high proportion of agricultural use because their effluents are mostly enriched by N, mainly in form of NO_3_^−^ derived from fertilizers that are not fixed to the exchangeable soil complex. In fact, wetlands have been highlighted as valuable ecosystems to mitigate the negative impacts of NO_3_^−^ excess because of their capacity to act as green filters (Álvarez-Rogel et al. 2016). On the other hand, low depth and standing water at S8 and S12 stations can also promote evaporate process, causing an increase in the concentration of pollutants. Also, all samples had nitrite (NO_2_^−^) and ammonia (NH_3_) values well below the FAO (1973), as shown in Table S8. The maximum NO_2_^−^ (0.038 mg/l) and NH_3_ (1.58 mg/l) value was also measured at the S8 station. Environmental and health concerns associated with different forms of nitrogen in water can be classified as human health, aquatic life toxicity, and eutrophication. Exposure to nitrate and in some cases nitrite polluted water has notably contributed to methemoglobinemia in infants. According to the Safe Drinking Water Act standard, known as a maximum contaminant level (MCL), established by the US Environmental Protection Agency (EPA), the nitrate and nitrite concentrations in the wetland water (except at S8 station for nitrate) were below 10 mg/l and 1 mg/l, respectively.

The highest concentration TP (0.2 mg/1) was found in the water of the S12 station due to receiving effluents from agricultural lands, and the lowest value was measured 0.02 mg/l in the water of S1 station, which is located close to Sabz Ali spring. Both total phosphorus (TP) and phosphate (PO_4_^3−^) values did not differ significantly between the different stations although TP concentration at all stations was twice high as that in the PO_4_^3−^ concentration, as shown in Fig. S11. The PO_4_^3−^concentration ranged between 0.01 mg/l and 0.14 mg/l with a mean value of 0.05 mg/1 (Table S8). Moreover, the PO_4_^3−^ concentration at all sampling points was below the recommended limit of 2 mg/l (FAO 1973). Increasing the PO_4_^3−^ concentration in water can cause accelerated growth of phytoplankton (algal blooms) and pose a threat to water eutrophication in the wetland. Nuisance algal growths are not uncommon in surface waters below the low reference level (0.1 mg/l) for P by recommended water quality criteria (US EPA 2002), however, the results of this study suggested that the P concentration at the S12 station is more than the reference level and can cause negative impacts in a long time on the wetland health. Zhang et al. (2003) found that in TP value between 0.16 and 0.25 mg/l, the water state changes from fresh to turbid resulting in a significant decrease in the submerged vegetation. There are various sources of P, both natural (such as soils, rocks and atmospheric precipitation) and human (including agricultural fertilizers, municipal and industrial wastewater) origins. Apatite [Ca_5_(PO_4_)_3_(F,Cl,OH)] is the most abundant naturally occurring P containing mineral in the Earth’s crust. The world’s main source of phosphatic fertilizer is rock phosphate, a naturally occurring P-rich sedimentary or igneous rock containing about 5–13% P (Nieder et al. 2018). The major external source of P in the water of Hashilan wetland is agricultural runoff.

To evaluate the trophic status of a water column there are no fixed or perfect assessment criteria. However, the general parameters to evaluate the trophic stage or eutrophication include dissolved oxygen concentration, water transparency, algal chlorophyll, and total nutrient concentration (P and N). In general, it is convenient to associate the trophic status in terms of nutrient concentration as this is the key factor controlling the eutrophication process (Bhagowati and Ahamad 2019). The general guideline for N and P concentrations indicating the different trophic states of the water column are given in Table S9. The eutrophication or red tide occurs when total nitrogen and phosphorus concentrations exceed 300 μg/l and 20 μg/l, respectively (Yang et al. 2008). According to the finding of Richardson et al. (2007), threshold protective of total phosphorus for all trophic levels would best be defined as a threshold zone at 12–15 μg/l, and exceeding surface water TP threshold value of 15 μg/l can cause an ecological imbalance of algal, macrophytes and other aquatic organisms. For data evaluation purposes nutrient levels in the Hashilan wetland are compared to the trophic status guidelines (Table S9). According to the trophic status classification scheme, based on the TP levels, the S2, S3, and S8 stations were at a eutrophic state. In the S1 station, the TP content was 0.02 mg/l and was at a mesotrophic state and the water quality of the S5 and S12 stations was hypertrophic level. Based on the mean TP concentration (0.08 mg/l), the water quality of the Hashilan wetland was at a eutrophic state. Concentrations of TP higher than 30 µg /l are generally considered favorable for eutrophication in freshwater ecosystems, provided that inorganic nitrogen or other nutrients are not limiting (De Villiers 2007). In addition, minimum and maximum TN concentrations indicate that all of the Hashilan wetland monitoring stations have come into hypertrophic conditions, i.e., TN values exceeding 2000 µg/l according to the trophic status classification scheme (Table S8 and S9). Our results indicated that TP and TN concentrations in Hashilan wetland exceeded the thresholds for algae blooms. On the other hand, increased levels of the nutrient towards the downstream of the wetland possibly reflect the intensity of human activities within the reaches of the catchment.

The ratio of N:P in the water body (referred to as the “Redfield ratio”) is an important indicator of which nutrient is limiting eutrophication. The high ratio of N:P which is greater than 16 indicates that P is most likely a limiting nutrient to plant growth (Zheng et al. 2019). The N:P ratio in all the water samples was higher than the Redfield ratio of 16, and was thus P limited. This coincided with the high inputs of N at this wetland (Fig. S12). Also, the N:P ratio shows that in Hashilan wetland, like most other freshwater ecosystems, is commonly P limited. In fact, phosphorus is the main limiting nutrient for the primary production of phytoplankton in many freshwater systems, while nitrogen is generally limiting in marine environments (Howarth and Marino 2006). Although excessive TP and TN in water are considered as the only factors inducing water eutrophication, nutrient enrichment is only necessary but not an adequate condition for the algal bloom. Water eutrophication can occur quickly when all of the influencing factors involving slow current velocity, excessive TP and TN, biodiversity and microbial activity, and temperature and other environmental factors are favorable (Yang et al. 2008).

## Conclusion

This study focused on the distribution, fate, ecological risk assessment, and source apportionments of heavy metal(loid)s in sediments of Hashilan wetland. In this research, several methods for pollution and risk assessment were used, in order to best interpret the pollution state of water and sediments of the wetland. The results showed that the highest values of the investigated elements belonged to farmlands that are located near the road. However, only the mean concentrations of Ni, Cr, and Cu in sediment samples were higher than those in the world-soil average. However, Ni and Cr were moderately enriched at most stations. The moderate and significant enrichments of Mo and Cu in sediments were measured at some sampling sites where agricultural runoff enters the wetland. Due to the moderate and significant enrichments of the mentioned elements, all stations were moderately and severely polluted. Among the several methods of risk assessment, the TRI equation better presented the toxicity risk of heavy metals regarding the results of the MPI index which shows more than half of the stations pose considerable to moderate environmental risk. The highest risk contribution belonged to the Ni element.

According to the EF and two multivariate receptor models, Pb, Zn, Ni, Co, As, Mn, Cr, Al Sc, and Cd were mainly originated from natural sources while Mo and Cu were derived from mixed natural and agricultural activities. High concentrations of Ni and Cr are related to the Ophiolite sequences of the Zagros fold-and-thrust belt which along with Cu and Mo have contaminated the sediments of most stations. Also, low pollution of TPH was observed in Hashilan sediment samples, proposing the impact of anthropogenic sources on the TPH values is low. The results indicated that the surface water of a few stations are contaminated with Zn, Cu, and Cd. However, the ecological risks of these elements to the aquatic biota were negligible. Also, downstream stations indicated higher phosphate and nitrate concentrations compared to the other sites due to the entrance of agricultural effluents in this part of the wetland. According to the mean TP and TN concentrations, the water quality of the wetland was at eutrophic and hypertrophic status, respectively. Totally, monitoring and urgent actions must be done to reduce the input of nutrients into the water body.

## Supplementary Information

Below is the link to the electronic supplementary material.

Supplementary file1 (DOCX 2.01 MB)

## Data Availability

All data generated or analyzed during this study are included in this published article [and its supplementary information files].
