# Supplementary material for: Eutrophication and sediment–water exchange of total petroleum hydrocarbons and heavy metals of Hashilan wetland, a national heritage in NW Iran
Source: Environ Sci Pollut Res Int. 2021 Dec 19;29(18):27007–25. doi: 10.1007/s11356-021-17937-x (PMC8989912; doi:10.1007/s11356-021-17937-x)
Supplement: Supplementary file 1 — Supplementary file1 (DOCX 2.01 MB) [file 11356_2021_17937_MOESM1_ESM.docx]

**Eutrophication and sediment-water exchange of total petroleum hydrocarbons and heavy metals of Hashilan wetland, a national heritage in NW Iran**

Sajjad Abbasi ^a,b*^, Sara Sheikh Fakhradini ^a^, Nematollah Jafarzadeh ^c^, Pooria Ebrahimi ^d^, Shirin Yavar Ashayeri ^a^

^a^ Department of Earth Sciences, College of Science, Shiraz University, Shiraz, 71454, Iran

^b^ Department of Radiochemistry and Environmental Chemistry, Faculty of Chemistry, Maria Curie-Skłodowska University, Lublin 20-031, Poland

^c^ Environmental Technologies Research Center, Ahvaz Jundishapur University of Medical Sciences, Ahvaz, Iran

^d^ Department of Earth, Environmental and Resources Sciences, University of Naples Federico II, 80126 Naples, Italy

* Corresponding authors.

a Department of Earth Sciences, College of Science, Shiraz University, 71454, Shiraz, Iran.

b Department of Radiochemistry and Environmental Chemistry, Faculty of Chemistry, Maria Curie-Skłodowska University, Lublin 20-031, Poland

E-mail address: [sajjad.abbasi.h@gmail.com](mailto:sajjad.abbasi.h@gmail.com); [sajjad.abbasi@shirazu.ac.ir](mailto:sajjad.abbasi@shirazu.ac.ir)

**2.4. Data analysis**

**2.4.1. Enrichment factor (*EF*)**

Enrichment factor was conducted to differentiate between the proportion of anthropogenic and geogenic sources of heavy metals in sediments (Ahamad et al., 2020; Anbuselvan and Sridharan, 2018; Looi et al., 2019). This equation normalizes the elements using a reference metal such as Sc, Al, and Fe to determine the PTEs contamination level in sediment environment comprehensively (Islam et al., 2018; Jahan and Strezov, 2018; Loska et al., 1997). This index is calculated as follows:

$EF=\frac{\left( \frac{C_{i}}{C_{ref}} \right)_{s}}{\left( \frac{C_{i}}{C_{ref}} \right)_{b}}$ (1)

Where *C_i_* is the relative value of *i* element, *C_ref_* is a natural background element and s and b illustrate the measured sample and background values, respectively. In this study, Sc was selected as the reference metal to normalize PTE contents in sediments (Abbasi et al., 2019; Hamdoun et al., 2015). The five classes of EF are as follows: deficiency to low enrichment (< 2), moderate enrichment (2-5), significant enrichment (5-20), very high enrichment (20-40), and extremely high enrichment (> 40) (Bastami et al., 2018; Duodu et al., 2016).

**2.4.2. Geoaccumulation index (*I_geo_*)**

The geoaccumulation index (I_geo_) proposed by Müller (1969), evaluates the contamination degree of PTEs in sediments (Han et al., 2017; Wu et al., 2020) which is estimated as:

$I_{geo}={log}_{2} (\frac{C_{i}}{{1.5\times B}_{i}})$ (2)Where I_geo_ is the geo-accumulation index of a sample site; *C_i_* is the intended metal(liod) concentration, *B_i_* is the background content of the intended metal(liod), and 1.5 is considered as the correction factor for the background data due to lithologic effects (Harikrishnan et al., 2017; Sojka et al., 2019). The seven classes of I_geo_ are as follows: no contamination; (I_geo_ ≤0), light to moderate (II); 0<I_geo_≤1, moderate (III); 1< I_geo_ ≤ 2, moderate to heavy (IV); 2 < I_geo_ ≤ 3, heavy (V); 3 < I_geo_ ≤ 4, heavy to extremely serious (VI); 4< I_geo_ ≤ 5, and extremely serious (VII); I_geo_ > 5 (Kuerban et al., 2020; Xiao et al., 2019).

**2.4.3. Contamination factor *(Cf)***

Contamination factor as an effective index investigates the contamination status and impact of PTEs on the sediment environment which is calculated by dividing metal content in a specified sediment sample to the reference content of the same metal (Pang et al., 2015; Zhao et al., 2018). As a limitation, the metals proportion through the lithologic and sedimentary processes is not considered in CF equation (Brady et al., 2015; Duodu et al., 2016). However, the Cf formula is calculated as follows:

$Cf=\frac{C_{i}}{C_{b}}$ (3)

Where *C_f_* is contamination factor, *C_i_* is the concentration of the intended metal in a sediment sample and *C_b_* is the background concentration of the corresponding metal. To determine the environmental pollution, four classes of Cf is described as follows (Anbuselvan and Sridharan, 2018; Hakanson, 1980): low contamination (<1), moderate contamination (1-3), considerable contamination (3-6) and very high contamination (> 6).

**2.4.4. Nemerow pollution index (*NPI*) and modified pollution index (*MPI*)**

Multi-element pollution indices developed to estimate water and sediment qualities as a result of the restriction of single element indices (Brady et al., 2015; Duodu et al., 2016). Thus, multi-metal pollution can be calculated by Nemerow pollution index (NPI) and modiﬁed pollution index (MPI) (Duodu et al., 2016; Hu et al., 2019). Modified pollution index (MPI) is an improvement of the Nemerow pollution index (NPI) and uses enrichment factors instead of contamination factors in its calculation. Also, MPI takes into account the background concentrations and the complex, non-conservative behavior of sediments. Moreover, the sediment qualification threshold of MPI was adjusted to give a more accurate qualification of sediment contamination. Therefore, MPI is unlikely to overestimate sediment contamination unlike the low trigger value of 3 used for NPI (Brady et al., 2015; Duodu et al., 2016). The calculation of these indices have been expressed as follows:

$MPI=\sqrt{\frac{{({EF}_{average})}^{2}+{({EF}_{max})}^{2}}{2}}$ (4)

*MPI*, *EF_average_* and *EF_max_* indicate modified pollution index, average of PTEs enrichment factors and maximum enrichment factor in a site, respectively.

$NPI=\sqrt{\frac{{CF}^{2}+{CF}_{max}^{2}}{2}}$ (5)

*NPI*, *CF* and *CF_max_* denote Nemerow pollution index, arithmetic mean of PTEs contamination factors and the maximum contamination factor in a site, respectively. Thresholds for
sediment quality classification of these indices are summarized in Table S4. Also, water quality based on this index classifies into three levels: NPI < 0.7 shows uncontaminated water, 0.7 < NPI < 1 represents that water pollution risk is probable, and NPI >1 indicates water has been contaminated seriously (Zhang et al., 2017).

**2.4.5. Heavy metal toxic load (*HMTL*)**

The heavy metal toxic load (HMTL) estimates the amount of metal(loid)s found in the water that may influence the human health. This index not only determines the amount of treatment required to purify the water for human use but also helps to specify an effective refinement and management plan, which is calculated by the following formula (Kumar et al., 2019; Proshad et al., 2020; Saha and Paul, 2019):

$HMTL= \sum_{i=1}^{n} C\times HIS$ (6)

Where *C* is the content of metal(loid), *n* is the number of metal(loid)s, and *HIS* is the hazard intensity score, which is based on the frequency of occurrence of the hazardous substance at the National Priorities List (NPL) sites prepared by ATSDR (2017), toxicity level of these substances, and potential for human exposure.

**2.4.6. Potential ecological risk index (*RI*) and modified ecological risk index (*MRI*)**

Potential risk of contaminant agents to the biological community is calculated via the method of potential ecological risk index (RI) developed by Hakanson (1980). This index helps to specify the contaminant agents and polluted situations in the water and sediment of the study area (Maanan et al., 2015; Proshad et al., 2020; Tang et al., 2013) which is defined as follows:

$RI=\sum_{i=1}^{n} {Er}^{i}=\sum_{i=1}^{n} {Tr}^{i}*{CF}^{i}$ (7)

*CF^i^* is contamination factor of *i_th_* element, and *Tr^i^* shows biological toxic response coefficient of *i_th_* element which is determined based on the individual PTEs toxicity and environmental sensitivity to them (Maanan et al., 2015). *Tr^i^* values for Cd, Cu, Co, Ni, Pb, Zn, Mn, and Cr are 30, 5, 5, 5, 5, 1 ,1 and 2, respectively (Hakanson, 1980; Tang et al., 2013; Vu et al., 2017). *Er^i^* is considered as the potential ecological risk factor of *i_th_* element, and potential ecological risk is obtained by sum of Er^i^.

For considering the lithogenic and sedimentary inputs, contamination factor could be replaced with enrichment factor; thus, ecological risk evaluation is modified as the following equation (Duodu et al., 2016):

$MRI=\sum_{i=1}^{n} {Er}^{i}=\sum_{i=1}^{n} {Tr}^{i}*{EF}^{i}$ (8)

*MRI*, *EF^i^* and *Tr^i^* represent modified potential ecological risk, enrichment factor of *i_th_* element and biological toxic response coefficient of *i_th_* element, respectively. Thresholds for water and sediment ecological risk classification are presented in Table S4.

**2.4.7. Sediment quality guidelines (SQGS)**

Sediment quality guidelines (SQG's) are applied to specify contamination and toxicity status in sediments (Costa-Böddeker et al., 2017; Ustaoğlu and Islam, 2020). To estimate the effects of each metal(loid), threshold effect level (TEL), and probable effect level (PEL) were proposed by McDonald et al. (2000) to predict the toxicity status in sediments. The concentrations below the TEL display rarely adverse effects, while concentrations above the PEL expect to show adverse effects (Liu et al., 2018; Ustaoğlu and Islam, 2020). Multiple pollutants display a more realistic toxicity compared to individual contaminant. Therefore, to evaluate the potential ecological risk of multiple metal(loid)s on aquatic biota, the mean PEL quotient was computed for each position according to the following formula (Gu, 2018; Ji et al., 2019):

$mean PEL quotient=\sum_{i=1}^{n} \frac{(\frac{C_{i}}{{PEL}_{i}})}{n}$ (9)

Where the *C_i_* is the content of metal(loid) *i* in sediments, the PEL_i_ is the guideline value for the metal(loid) *i*, and *n* is the number of metal(loid)s. Mean PEL quotient (mPELQ) values of < 0.1, 0.11–1.5, 1.51–2.3 and > 2.3 define four classes of toxicity probability as low (10%), (medium-low) 25%, medium-high (50%) and high (76%) priority sites, respectively, for the biota in sediments.

**2.4.8. Toxic units (*TUs*)**

Toxic units were suggested by Pedersen et al (1998) to estimate the toxic effects of heavy metals in sediments. Also, *TUs* normalize the toxicities caused by different PTEs to compare their relative effects (Zhang et al., 2016a). This index is described as the concentration of each heavy metal relative to the corresponding PEL value and sum of toxic units at each sampling sites illustrate the level of potential acute toxicity of multiple heavy metals at that position. The *ΣTU* index is calculated as the follows (Ji et al., 2019; Niu et al., 2020):

$\sum TU= \sum_{i=1}^{n} \frac{C_{s}^{i}}{C_{PEL}^{i}}$ (10)

Where *n* is the number of heavy metals*, C_s_^i^* is the intended metal(loid) value in a sediment sample, *C^i^_PEL_* is the PEL content of the corresponding metal(loid). The toxicity level of ΣTUs are classified as: low toxicity level; ΣTUs<4, moderately toxicity level; 4≤ ΣTUs ≤6, and heavily toxicity level; ΣTUs＞6 (Ustaoğlu and Islam, 2020).

**2.4.9. Toxic risk index (*TRI*)**

Toxic risk index **(**TRI) was suggested by Zhang et al (2016b) based on TEL and PEL contents to estimate the integrated toxicity risk. The TRI for each metal(loid) is calculated by the following equation:

$TRI=\sum_{i=1}^{n} {TRI}_{i}=\sqrt{\frac{({C_{s}^{i}/C_{TEL}^{i})}^{2}+({C_{s}^{i}/C_{PEL}^{i})}^{2}}{2}}$ (11)

Where *n* is the number of heavy metals*, C_s_^i^* is the concentration of the intended metal(loid) in a sediment sample, *C^i^_TEL_* and *C^i^_PEL_* are the TEL and PEL values of the corresponding metal(loid), respectively (Ustaoğlu and Islam, 2020). According to TRI calculation, the level of toxicity risks are categorized into five classes: no toxic risk; TRI *≤* 5, low toxic risk; 5 < TRI *≤* 10, moderate toxic risk; 10 < TRI *≤* 15, considerable toxic risk; 15 < TRI *≤* 20, and very high toxic risk; TRI > 20 (Sojka et al., 2019; Zeng et al., 2020).

**2.4.10. Distribution coefficient (*K_p_*) between water and sediment**

To discover the interaction of PTEs between water and sediment phases, partition coefficient (*K_p_*) was applied (Duarte et al., 2014; Feng et al., 2017). This index determines a quantitative content for the partitioning of metal concentration between water and sediment phases. Distribution coefficient (K_p_) formula is represented as follows (Zeng et al., 2019):

$Kp=\frac{C_{s}}{C_{d}}$ (12)

Where *C_s_* is the metal value in sediment phase (μg/g) and *C_d_* is the dissolved metal value in water phase (μg/L). The Pearson correlation coefficient was used via Statistical Package for Social Science (SPSS) to recognize the effect of some physico-chemical parameters on metal partition coefficients.

**2.4.11. Source identification**

Statistical computations of data were conducted using SPSS 23.0 and Excel 2016 software for Windows. Descriptive statistical parameters such as mean and standard deviation (SD) of the PTEs concentrations in water and sediments were computed to study the distribution of the variables. The data normality was investigated using the Shapiro-Wilk test (p<0.05). Since the concentrations of some heavy metals were below the corresponding detection limits in more than 50% of samples, they were deleted from the data before statistical analysis. Also, in order to conduct the statistical analysis, the values of heavy metals below the detection limit were replaced with 75% of the corresponding detection limits. Spearman’s correlation analysis was applied to evaluate the significant relationships between PTEs and sediment properties due to non-normal distribution of some variables. Also, Levene's test for equality of variances was performed, which shows whether the variances from soil and sediment zones are different. A value of greater than 0.05 demonstrates that the assumption of equal variances is acceptable. PCA-APCS-MLR and PMF models were applied to identify the PTEs sources which are described as follows.

**2.4.11.1. PCA-APCS-MLR model**

Principal component analysis (PCA) as an efficient tool is performed to specify the source of PTEs in the study area by converting a large set of data to small independent variables (Yang et al., 2020). Applicability data for PCA is determined by Kaiser-Meyer-Olkin (KMO) and Bartlett’s Sphericity test (Sheikh Fakhradini et al., 2019). The interpretation of primarily PCA factors may be difficulty done; thus, varimax rotation is performed to achieve a simple structure of factors by redistribution and polarization of the original PCA loadings (Zhang et al., 2020). Meaningful factors is selected via eigenvalues greater than 1.0 (Zhang et al., 2018).

To perform MLR method, PCA factor scores and the total concentrations of PTEs are used as independent and dependent variables, respectively. Then, the obtained standardized regression coefficients are used to determine proportional share of each source (Salim et al., 2019). The detailed steps of PCA-MLR model have been described by Larsen and Baker (2003).
The absolute principal component scores (APCS) model not only qualitatively specifies the load species of each pollution source, but also quantitatively specifies the mean source contribution to its metal(loid)s and the portion rate at each sampling site (Jin et al., 2019; Yang et al., 2020). This model has been explained in detail by Thurston and Spengler (1985). To assess the APCS of each metal(liod) at each sampling site, the factor score of *Z_0_* is subtracted from the factor score of *Z_ik_* (the true sample) which is calculated by the following formulas (Jin et al., 2019; Zhang et al., 2020):

${{(Z}_{i})}_{j}=\frac{C_{ij}-\bar{C}_{j}}{\sigma_{j}}=-\frac{\bar{C}_{j}}{\sigma_{j}}$ (13)

${{(Z}_{0})}_{j}=\frac{0-\bar{C}_{j}}{\sigma_{j}}=-\frac{\bar{C}_{j}}{\sigma_{j}}$ (14)

${{(A}_{0})}_{k}= \sum_{j=1}^{j} S_{jk}\times{{(Z}_{0})}_{j}$ (15)

${APCS}_{ik}= {{(A}_{z})}_{ik}-({A_{0})}_{k}$ (16)

Where *C_ij_* is the value of *j_th_* species in *i_th_* sample; *C_j_* and *σ_j_* are the average concentration and standard deviation of *j_th_* species, respectively; *(A_0_)_k_* are the scores of zero points; *S_jk_* is coefficient of principal component scores; *(A_z_)_ik_* are composite scores of principal components.

Then, the absolute principal component scores (APCS) obtained were used to estimate the source portions of the metal(liod)s via multiple linear regression (MLR) equation which was expressed as follows (Guan et al., 2019; Lv, 2019):

$M_{i}=\varsigma_{0}+ \sum_{k=1}^{p} \varsigma_{k}{APCS}_{ki}$ (17)

Where *M_i_* is the mean concentration of *i_th_* species; *ς_0_* is the constant term of multiple regressions for *i_th_* species; *ς_k_* is the coefficient of multiple regression of *k_th_* source. *APCS_ki_* is
the absolute principle component score for *k_th_* source in *i_th_* sample. And *ς_k_ APCS*_ki_ is the portion of the *p_th_* factor to *M_i_.*

**2.4.11.2. PMF model analysis**

Positive Matrix Factorization (PMF) model is performed to partition the portion of different sources to metal(loid)s values in the sediment (Zhang et al., 2020). All variables in this technique are given an individual weights, and factor loadings have no negative values which provide a better description of results compared to the PCA analysis (Han et al., 2017; Liu et al., 2019; Vu et al., 2017). In PMF model, n and m as the number of samples and species constitute data matrix X which is factorized into two matrices G (n p) and F (p m) according to Eq. (18) (Comero et al., 2014; Karanasiou et al., 2009):

$c_{ij}= \sum_{k=1}^{p} g_{ik}f_{kj}+e_{ij}$ (18)

Where *C_ij_* is the value of *j_th_* metal(loid) in *i_th_* sample, *p* is the number of factors, *G_ik_* is the contribution of *k_th_* source to *i_th_* sample, *F_kj_* is the value of *j_th_* metal(loid) from *k_th_* source, and *e_ij_* is the residual error matrix.

The PMF model minimize the objective function Q in order to derive the factor contributions and profiles, which is presented as the follows (Kuerban et al., 2020; Liu et al., 2019):

$Q=\sum_{i=1}^{n} \sum_{j=1}^{m} \left( \frac{e_{ij}}{u_{ij}} \right)^{2}$ (19)

Where m is the number of metal(loid)s, n is the number of samples, and *u_ij_* is the uncertainty of *j_th_* metal(loid) in *i_th_* sample which is specified based on the intended metal(loid) value (*C_ij_*), error fraction and species-specific method detection limit (CMDL). If the content of metal(loid) is above the corresponding MDL value, the uncertainty will be calculated by Eq. (20). While, the Eq. (21) is applied when the mentioned metal(loid) value is below its corresponding MDL value (Cheng et al., 2020; Yuanan et al., 2020). However, the detailed information about Positive Matrix Factorization (PMF) could be found in the work Paatero and Tapper (1994) and Paatero (1997).

$Unc=\sqrt{{(Errorfraction\times c_{ij})}^{2}+{(0.5\times MDL)}^{2}}$ (20)

$Unc=\frac{5}{6}\times MDL$ (21)

**Table S1**

Location and general characteristics of sediment and water samples.

**TableS2**

Levene's test for variance tests equality of heavy metal(loid)s in soil and sediments of the Hashilan wetland.

**Table S3**

Heavy metal(loid)s concentrations in sediments of the present study and other similar studies (mg/Kg).

**Table S4**

Thresholds for sediment quality classification for multi-element indices and grading standards of potential and modified ecological risk index (Duodu et al., 2016).

**Table S5**

The Spearman correlation between the physiochemical parameters and heavy metal(loid)s concentrations in sediment.

**Table S6**

Statistical summary of partition coefficients (log *K_p_* in L/kg) of heavy metal(loid)s obtained from the Hashilan wetland.

**Table S7**

Correlation coefficients of the sediment-water partition coefficients (*K_p_*) of the trace elements with influential factors.

**Table S8**

Summary statistics of Phosphorus and nitrogen compounds in water samples of Hashilan wetland.

**Table S9**

The concentrations of total phosphorus (TP) and total nitrogen (TN) in different Eutrophic states of water.

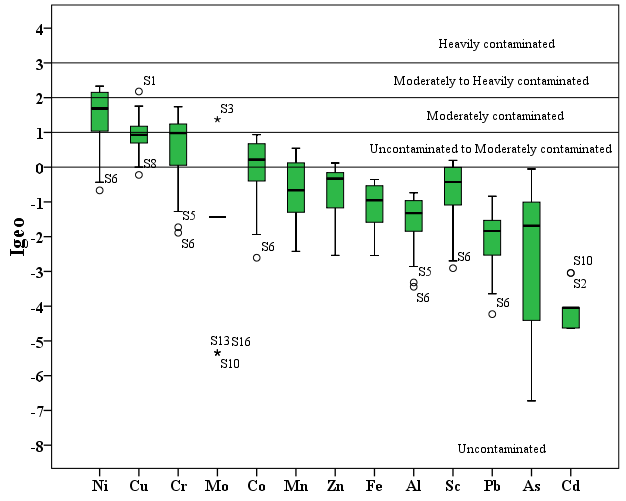


**Fig. S1.** Geoaccumulation index, I_geo_, of heavy metal(loid)s in sediments of the Hashilan wetland.


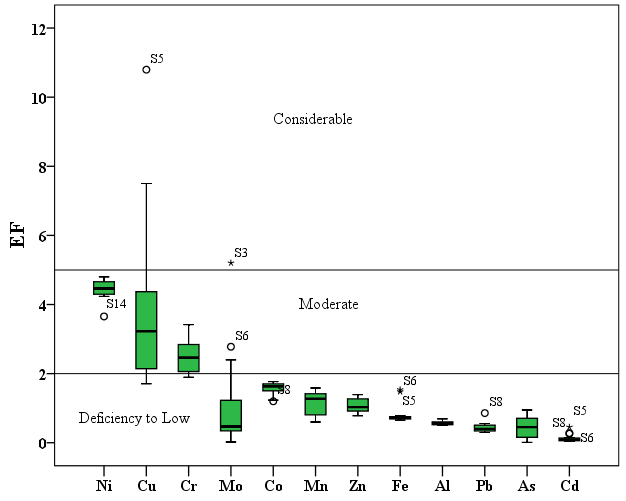


**Fig. S2.** Enrichment factor, EF, of heavy metal(loid)s in sediments of the Hashilan wetland.


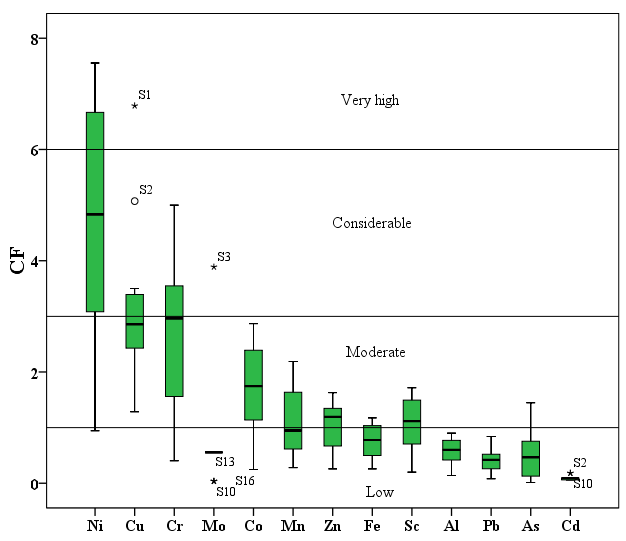


**Fig. S3.** Contamination factor, CF, of heavy metal(loid)s in sediments of the Hashilan wetland.

**Fig. S4.** Sediment quality assessment by multi-element indices (NPI: Nemerow pollution index and MPI: modified pollution index). (See Table S2 for sediment classification dash lines).

**Fig. S5.** Water quality assessment by Nemerow pollution index (NPI).

**Fig. S6.** Assessment ecological risk posed by heavy metal(loid)s in surface sediments of Hashilan wetland using potential ecological risk index (RI) and modified ecological risk index (MRI). (See Table S2 for sediment classification dash lines).

**Fig. S7.** The TUs and ΣTUs values of the seven heavy metal(loid)s in surface sediments of Hashilan wetland.

**Fig. S8.** TRI values of the seven heavy metal(loid)s in surface sediments of Hashilan wetland

**Fig. S9.** Assessment ecological risk posed by heavy metal(loid)s in surface water of Hashilan wetland using potential ecological risk index (RI).

**Fig. S10.** The concentration of nitrate (NO_3_^-^) in water sampling stations.

**Fig. S11.** The concentrations of total phosphorus (TP) and phosphate (PO_4_^3-^) in water sampling stations.


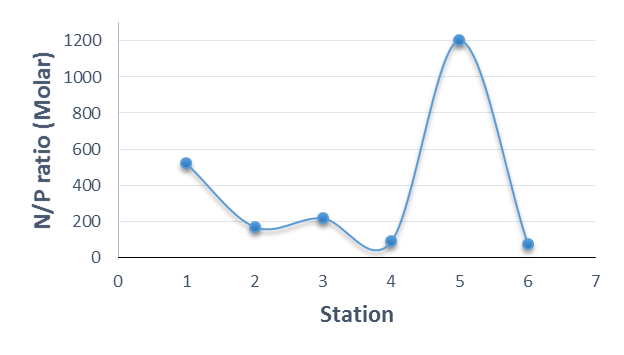


**Fig. S12.** The variations of N/P ratio at water sampling stations in Hashilan wetland.

**Reference**

Abbasi, S., Keshavarzi, B., Moore, F., Shojaei, N., Sorooshian, A., Soltani, N., Delshab, H., 2019. Geochemistry and environmental effects of potentially toxic elements, polycyclic aromatic hydrocarbons and microplastics in coastal sediments of the Persian Gulf. Environmental Earth Sciences 78, 492.

Ahamad, M.I., Song, J., Sun, H., Wang, X., Mehmood, M.S., Sajid, M., Su, P., Khan, A.J., 2020. Contamination Level, Ecological Risk, and Source Identification of Heavy Metals in the Hyporheic Zone of the Weihe River, China. International journal of environmental research and public health 17, 1070.

Anbuselvan, N., Sridharan, M., 2018. Heavy metal assessment in surface sediments off Coromandel Coast of India: Implication on marine pollution. Marine Pollution Bulletin 131, 712–726.

ATSDR (Agency for Toxic Substances and Disease Registry), 2017. Agency for toxic substances and disease registry, substance priority list. Available at https://www.atsdr.cdc.gov/spl.index.html#2019spl.

Bastami, K.D., Neyestani, M.R., Molamohyedin, N., Shafeian, E., Haghparast, S., Shirzadi, I.A., Baniamam, M., 2018. Bioavailability, mobility, and origination of metals in sediments from Anzali Wetland, Caspian Sea. Marine Pollution Bulletin 136, 22–32.

Brady, J.P., Ayoko, G.A., Martens, W.N., Goonetilleke, A., 2015. Development of a hybrid pollution index for heavy metals in marine and estuarine sediments. Environmental monitoring and assessment 187, 306.

Cheng, W., Lei, S., Bian, Z., Zhao, Y., Li, Y., Gan, Y., 2020. Geographic distribution of heavy metals and identification of their sources in soils near large, open-pit coal mines using positive matrix factorization. Journal of hazardous materials 387, 121666.

Comero, S., Vaccaro, S., Locoro, G., De Capitani, L., Gawlik, B.M., 2014. Characterization of the Danube River sediments using the PMF multivariate approach. Chemosphere 95, 329–335.

Costa-Böddeker, S., Hoelzmann, P., Huy, H.D., Nguyen, H.A., Richter, O., Schwalb, A., 2017. Ecological risk assessment of a coastal zone in Southern Vietnam: Spatial distribution and content of heavy metals in water and surface sediments of the Thi Vai Estuary and Can Gio Mangrove Forest. Marine pollution bulletin 114, 1141–1151.

Duarte, B., Silva, G., Costa, J.L., Medeiros, J.P., Azeda, C., Sá, E., Metelo, I., Costa, M.J., Caçador, I., 2014. Heavy metal distribution and partitioning in the vicinity of the discharge areas of Lisbon drainage basins (Tagus Estuary, Portugal). Journal of sea research 93, 101–111.

Duodu, G.O., Goonetilleke, A., Ayoko, G.A., 2016. Comparison of pollution indices for the assessment of heavy metal in Brisbane River sediment. Environmental pollution 219, 1077–1091.

Sheikh Fakhradini, S., Moore, F., Keshavarzi, B., Lahijanzadeh, A., 2019. Polycyclic aromatic hydrocarbons (PAHs) in water and sediment of Hoor Al-Azim wetland, Iran: a focus on source apportionment, environmental risk assessment, and sediment-water partitioning. Environmental monitoring and assessment 191, 233.

Feng, C., Guo, X., Yin, S., Tian, C., Li, Y., Shen, Z., 2017. Heavy metal partitioning of suspended particulate matter–water and sediment–water in the Yangtze Estuary. Chemosphere 185, 717–725.

Gu, Y.G., 2018. Heavy metal fractionation and ecological risk implications in the intertidal surface sediments of Zhelin Bay, South China. Marine pollution bulletin 129, 905–912.

Guan, Q., Zhao, R., Pan, N., Wang, F., Yang, Y., Luo, H., 2019. Source apportionment of heavy metals in farmland soil of Wuwei, China: comparison of three receptor models. Journal of Cleaner Production 237, 117792.

Hakanson, L., 1980. An ecological risk index for aquatic pollution control. A sedimentological approach. Water research 14, 975–1001.

Hamdoun, H., Van-Veen, E., Basset, B., Lemoine, M., Coggan, J., Leleyter, L., Baraud, F., 2015. Characterization of harbor sediments from the English Channel: assessment of heavy metal enrichment, biological effect and mobility. Marine pollution bulletin 90, 273–280.

Han, D., Cheng, J., Hu, X., Jiang, Z., Mo, L., Xu, H., Ma, Y., Chen, X., Wang, H., 2017. Spatial distribution, risk assessment and source identification of heavy metals in sediments of the Yangtze River Estuary, China. Marine Pollution Bulletin 115, 141–148.

Harikrishnan, N., Ravisankar, R., Chandrasekaran, A., Gandhi, M.S., Kanagasabapathy, K. V, Prasad, M.V.R., Satapathy, K.K., 2017. Assessment of heavy metal contamination in marine sediments of east coast of Tamil Nadu affected by different pollution sources. Marine pollution bulletin 121, 418–424.

Hu, J., Lin, B., Yuan, M., Lao, Z., Wu, K., Zeng, Y., Liang, Z., Li, H., Li, Y., Zhu, D., 2019. Trace metal pollution and ecological risk assessment in agricultural soil in Dexing Pb/Zn mining area, China. Environmental geochemistry and health 41, 967–980.

Islam, M.S., Hossain, M.B., Matin, A., Sarker, M.S.I., 2018. Assessment of heavy metal pollution, distribution and source apportionment in the sediment from Feni River estuary, Bangladesh. Chemosphere 202, 25–32.

Jahan, S., Strezov, V., 2018. Comparison of pollution indices for the assessment of heavy metals in the sediments of seaports of NSW, Australia. Marine pollution bulletin 128, 295–306.

Ji, Z., Zhang, H., Zhang, Y., Chen, T., Long, Z., Li, M., Pei, Y., 2019. Distribution, ecological risk and source identification of heavy metals in sediments from the Baiyangdian Lake, Northern China. Chemosphere 237, 124425.

Jin, G., Fang, W., Shafi, M., Wu, D., Li, Y., Zhong, B., Ma, J., Liu, D., 2019. Source apportionment of heavy metals in farmland soil with application of APCS-MLR model: a pilot study for restoration of farmland in Shaoxing City Zhejiang, China. Ecotoxicology and environmental safety 184, 109495.

Karanasiou, A.A., Siskos, P.A., Eleftheriadis, K., 2009. Assessment of source apportionment by Positive Matrix Factorization analysis on fine and coarse urban aerosol size fractions. Atmospheric Environment 43, 3385–3395.

Kuerban, M., Maihemuti, B., Waili, Y., Tuerhong, T., 2020. Ecological risk assessment and source identification of heavy metal pollution in vegetable bases of Urumqi, China, using the positive matrix factorization (PMF) method. PloS one 15, e0230191.

Kumar, V., Parihar, R.D., Sharma, A., Bakshi, P., Sidhu, G.P.S., Bali, A.S., Karaouzas, I., Bhardwaj, R., Thukral, A.K., Gyasi-Agyei, Y., 2019. Global evaluation of heavy metal content in surface water bodies: A meta-analysis using heavy metal pollution indices and multivariate statistical analyses. Chemosphere 236, 124364.

Larsen, R.K., Baker, J.E., 2003. Source apportionment of polycyclic aromatic hydrocarbons in the urban atmosphere: a comparison of three methods. Environmental Science & Technology 37, 1873–1881.

Liu, Q., Jia, Z., Li, S., Hu, J., 2019. Assessment of heavy metal pollution, distribution and quantitative source apportionment in surface sediments along a partially mixed estuary (Modaomen, China). Chemosphere 225, 829–838.

Liu, Q., Wang, F., Meng, F., Jiang, L., Li, G., Zhou, R., 2018. Assessment of metal contamination in estuarine surface sediments from Dongying City, China: use of a modified ecological risk index. Marine Pollution Bulletin 126, 293–303.

Looi, L.J., Aris, A.Z., Yusoff, F.M., Isa, N.M., Haris, H., 2019. Application of enrichment factor, geoaccumulation index, and ecological risk index in assessing the elemental pollution status of surface sediments. Environmental geochemistry and health 41, 27–42.

Loska, K., Cebula, J., Pelczar, J., Wiechuła, D., Kwapuliński, J., 1997. Use of enrichment, and contamination factors together with geoaccumulation indexes to evaluate the content of Cd, Cu, and Ni in the Rybnik water reservoir in Poland. Water, Air, and Soil Pollution 93, 347–365.

Lv, J., 2019. Multivariate receptor models and robust geostatistics to estimate source apportionment of heavy metals in soils. Environmental pollution 244, 72–83.

Maanan, M., Saddik, M., Maanan, M., Chaibi, M., Assobhei, O., Zourarah, B., 2015. Environmental and ecological risk assessment of heavy metals in sediments of Nador lagoon, Morocco. Ecological Indicators 48, 616–626.

Niu, Y., Jiang, X., Wang, K., Xia, J., Jiao, W., Niu, Y., Yu, H., 2020. Meta analysis of heavy metal pollution and sources in surface sediments of Lake Taihu, China. Science of The Total Environment 700, 134509.

Paatero, P., 1997. Least squares formulation of robust non-negative factor analysis. Chemometrics and intelligent laboratory systems 37, 23–35.

Paatero, P., Tapper, U., 1994. Positive matrix factorization: A non‐negative factor model with optimal utilization of error estimates of data values. Environmetrics 5, 111–126.

Pang, H.J., Lou, Z.H., Jin, A.M., Yan, K.K., Jiang, Y., Yang, X.H., Chen, C.T.A., Chen, X.G., 2015. Contamination, distribution, and sources of heavy metals in the sediments of Andong tidal flat, Hangzhou bay, China. Continental Shelf Research 110, 72–84.

Pedersen, F., Bjørnestad, E., Andersen, H.V., Kjølholt, J., Poll, C., 1998. Characterization of sediments from Copenhagen Harbour by use of biotests. Water Science and Technology 37, 233–240.

Proshad, R., Islam, S., Tusher, T.R., Zhang, D., Khadka, S., Gao, J., Kundu, S., 2020. Appraisal of heavy metal toxicity in surface water with human health risk by a novel approach: a study on an urban river in vicinity to industrial areas of Bangladesh. Toxin Reviews 1–17.

Saha, P., Paul, B., 2019. Assessment of heavy metal toxicity related with human health risk in the surface water of an industrialized area by a novel technique. Human and Ecological Risk Assessment: An International Journal 25, 966–987.

Salim, I., Sajjad, R.U., Paule-Mercado, M.C., Memon, S.A., Lee, B.Y., Sukhbaatar, C., Lee, C.H., 2019. Comparison of two receptor models PCA-MLR and PMF for source identification and apportionment of pollution carried by runoff from catchment and sub-watershed areas with mixed land cover in South Korea. Science of The Total Environment 663, 764–775.

Sojka, M., Jaskuła, J., Siepak, M., 2019. Heavy metals in bottom sediments of reservoirs in the lowland area of western Poland: concentrations, distribution, sources and ecological risk. Water 11, 56.

Tang, W., Zhao, Y., Wang, C., Shan, B., Cui, J., 2013. Heavy metal contamination of overlying waters and bed sediments of Haihe Basin in China. Ecotoxicology and environmental safety 98, 317–323.

Thurston, G.D., Spengler, J.D., 1985. A quantitative assessment of source contributions to inhalable particulate matter pollution in metropolitan Boston. Atmospheric Environment (1967) 19, 9–25.

Ustaoğlu, F., Islam, M.S., 2020. Potential toxic elements in sediment of some rivers at Giresun, Northeast Turkey: A preliminary assessment for ecotoxicological status and health risk. Ecological Indicators 113, 106237.

Vu, C.T., Lin, C., Shern, C.C., Yeh, G., Tran, H.T., 2017. Contamination, ecological risk and source apportionment of heavy metals in sediments and water of a contaminated river in Taiwan. Ecological indicators 82, 32–42.

Wu, J., Margenot, A.J., Wei, X., Fan, M., Zhang, H., Best, J.L., Wu, P., Chen, F., Gao, C., 2020. Source apportionment of soil heavy metals in fluvial islands, Anhui section of the lower Yangtze River: comparison of APCS–MLR and PMF. Journal of Soils and Sediments 1–14.

Xiao, H., Shahab, A., Li, J., Xi, B., Sun, X., He, H., Yu, G., 2019. Distribution, ecological risk assessment and source identification of heavy metals in surface sediments of Huixian karst wetland, China. Ecotoxicology and Environmental Safety 185, 109700.

Yang, Y., Yang, X., He, M., Christakos, G., 2020. Beyond mere pollution source identification: Determination of land covers emitting soil heavy metals by combining PCA/APCS, GeoDetector and GIS analysis. Catena 185, 104297.

Yuanan, H., He, K., Sun, Z., Chen, G., Cheng, H., 2020. Quantitative source apportionment of heavy metal (loid) s in the agricultural soils of an industrializing region and associated model uncertainty. Journal of Hazardous Materials 391, 122244.

Zeng, J., Han, G., Wu, Q., Tang, Y., 2019. Heavy metals in suspended particulate matter of the Zhujiang River, southwest China: contents, sources, and health risks. International journal of environmental research and public health 16, 1843.

Zeng, J., Han, G., Yang, K., 2020. Assessment and sources of heavy metals in suspended particulate matter in a tropical catchment, northeast Thailand. Journal of Cleaner Production 121898.

Zhang, G., Bai, J., Zhao, Q., Lu, Q., Jia, J., Wen, X., 2016a. Heavy metals in wetland soils along a wetland-forming chronosequence in the Yellow River Delta of China: levels, sources and toxic risks. Ecological Indicators 69, 331–339.

Zhang, H., Cheng, S., Li, H., Fu, K., Xu, Y., 2020. Groundwater pollution source identification and apportionment using PMF and PCA-APCA-MLR receptor models in a typical mixed land-use area in Southwestern China. Science of The Total Environment 741, 140383.

Zhang, Y., Chu, C., Li, T., Xu, S., Liu, L., Ju, M., 2017. A water quality management strategy for regionally protected water through health risk assessment and spatial distribution of heavy metal pollution in 3 marine reserves. Science of The Total Environment 599, 721–731.

Zhang, Z., Lu, Y., Li, H., Tu, Y., Liu, B., Yang, Z., 2018. Assessment of heavy metal contamination, distribution and source identification in the sediments from the Zijiang River, China. Science of the Total Environment 645, 235–243.

Zhang, Z., Wang, J.J., Ali, A., DeLaune, R.D., 2016b. Heavy metal distribution and water quality characterization of water bodies in Louisiana’s Lake Pontchartrain Basin, USA. Environmental monitoring and assessment 188, 628.

Zhao, Y., Xu, M., Liu, Q., Wang, Z., Zhao, L., Chen, Y., 2018. Study of heavy metal pollution, ecological risk and source apportionment in the surface water and sediments of the Jiangsu coastal region, China: a case study of the Sheyang Estuary. Marine pollution bulletin 137, 601–609.

Bai, J., Cui, B., Chen, B., Zhang, K., Deng, W., Gao, H., Xiao, R., 2011. Spatial distribution and ecological risk assessment of heavy metals in surface sediments from a typical plateau lake wetland, China. Ecological Modelling 222, 301–306.

Bastami, K.D., Neyestani, M.R., Molamohyedin, N., Shafeian, E., Haghparast, S., Shirzadi, I.A., Baniamam, M., 2018. Bioavailability, mobility, and origination of metals in sediments from Anzali Wetland, Caspian Sea. Marine Pollution Bulletin 136, 22–32.

Bonanno, G., Borg, J.A., Di Martino, V., 2017. Levels of heavy metals in wetland and marine vascular plants and their biomonitoring potential: a comparative assessment. Science of the Total Environment 576, 796–806.

Duodu, G.O., Goonetilleke, A., Ayoko, G.A., 2016. Comparison of pollution indices for the assessment of heavy metal in Brisbane River sediment. Environmental pollution 219, 1077–1091.

El‐Shazly, M.M., Omar, W.A., Edmardash, Y.A., Ibrahim, M.S., Elzayat, E.I., El‐Sebeay, I.I.A., Abdel Rahman, K.M., Soliman, M.M., 2017. Area reduction and trace element pollution in Nile Delta wetland ecosystems. African journal of ecology 55, 391–401.

Kehrig, H.A., Pinto, F.N., Moreira, I., Malm, O., 2003. Heavy metals and methylmercury in a tropical coastal estuary and a mangrove in Brazil. Organic Geochemistry 34, 661–669.

Mortazavi, S., Saberinasab, F., 2017. Heavy metals assessment of surface sediments in Mighan wetland using the sediment quality index. Ecopersia 5, 1761–1770.

Ramachandra, T. V, Sudarshan, P.B., Mahesh, M.K., Vinay, S., 2018. Spatial patterns of heavy metal accumulation in sediments and macrophytes of Bellandur wetland, Bangalore. Journal of environmental management 206, 1204–1210.

Richardson, C.J., King, R.S., Qian, S.S., Vaithiyanathan, P., Qualls, R.G., Stow, C.A., 2007. Estimating ecological thresholds for phosphorus in the Everglades. Environmental Science and Technology 41, 8084–8091. https://doi.org/10.1021/es062624w.

Sarkar, S.K., 2018. Trace Element Contamination in Surface Sediment of Sundarban Wetland, in: Trace Metals in a Tropical Mangrove Wetland. Springer, pp. 79–100.

Tam, N.F.Y., Wong, Y.S., 2000. Spatial variation of heavy metals in surface sediments of Hong Kong mangrove swamps. Environmental Pollution 110, 195–205.

Wang, J., Ye, S., Laws, E.A., Yuan, H., Ding, X., Zhao, G., 2017. Surface sediment properties and heavy metal pollution assessment in the Shallow Sea Wetland of the Liaodong Bay, China. Marine pollution bulletin 120, 347–354.

Weis, D.A., Callaway, J.C., Gersberg, R.M., 2001. Vertical accretion rates and heavy metal chronologies in wetland sediments of the Tijuana Estuary. Estuaries 24, 840–850.

Yang, X.E., Wu, X., Hao, H.L., He, Z.L., 2008. Mechanisms and assessment of water eutrophication. Journal of Zhejiang University: Science B. https://doi.org/10.1631/jzus.B0710626

Yavar Ashayeri, N., Keshavarzi, B., 2019. Geochemical characteristics, partitioning, quantitative source apportionment, and ecological and health risk of heavy metals in sediments and water: A case study in Shadegan Wetland, Iran. Marine pollution bulletin 149, 110495.

Zhang, H., Cui, B., Xiao, R., Zhao, H., 2010. Heavy metals in water, soils and plants in riparian wetlands in the Pearl River Estuary, South China. Procedia Environmental Sciences 2, 1344–1354.

Zhang, M., Cui, L., Sheng, L., Wang, Y., 2009. Distribution and enrichment of heavy metals among sediments, water body and plants in Hengshuihu Wetland of Northern China. Ecological engineering 35, 563–569.
